# Maternal Consumption of Ultra-Processed Foods-Rich Diet and Perinatal Outcomes: A Systematic Review and Meta-Analysis

**DOI:** 10.3390/nu14153242

**Published:** 2022-08-08

**Authors:** Walkyria O. Paula, Erika S. O. Patriota, Vivian S. S. Gonçalves, Nathalia Pizato

**Affiliations:** 1 Graduate Program in Human Nutrition, Faculty of Health Sciences, University of Brasilia, 70910-900 Brasilia, Brazil; 2 Graduate Program in Public Health, Faculty of Health Sciences, University of Brasilia, 70910-900 Brasilia, Brazil

**Keywords:** maternal diet, NOVA classification, perinatal outcomes

## Abstract

The consumption of ultra-processed food (UPF)-rich diets represents a potential threat to human health. Considering maternal diet adequacy during pregnancy is a major determinant for perinatal health outcomes, this study aimed to systematically review and meta-analyze studies investigating the association between maternal consumption of a UPF-rich diet and perinatal outcomes. Conducted according to the Preferred Reporting Items for Systematic Reviews and Meta-Analysis (PRISMA) guidelines, five electronic databases and gray literature using Google Scholar and ProQuest Dissertations and Theses Global were searched up to 31 May 2022. No restrictions were applied on language and publication date. Two reviewers independently conducted the study selection and data extraction process. Meta-analysis was conducted according to the random-effects model. In total, 61 studies were included in the systematic review and the overall population comprised 698,803 women from all gestational trimesters. Meta-analysis of cohort studies showed that maternal consumption of UPF-rich diets was associated with an increased risk of gestational diabetes mellitus (odds ratio (OR): 1.48; 95% confidence interval (CI): 1.17, 1.87) and preeclampsia (OR: 1.28; 95% CI: 1.15, 1.42). Neonatal outcomes showed no association. The overall GRADE quality of the evidence for the associations was very low. The findings highlight the need to monitor and reduce UPF consumption, specifically during the gestational period, as a strategy to prevent adverse perinatal outcomes.

## 1. Introduction

Significant metabolic and physiological changes occur during pregnancy, to support fetal growth and development [1]. Maternal diet quality is a major determinant for perinatal outcomes including hypertensive disorders, gestational diabetes, low birth weight, large gestational age, and preterm birth [2]. Furthermore, inadequate diet quality during pregnancy is associated with chronic diseases in later life such as type 2 diabetes mellitus, obesity, hypertension, and cardiovascular disorders [3]. 

Additionally to the evidence of the relationship between maternal diet quality and perinatal outcomes, several studies have reported high consumption of unhealthy and ultra-processed foods (UPFs) by pregnant women indicating a generally worse quality of diet [4,5,6,7]. 

The NOVA food classification system has been applied worldwide to evaluate the impact of modern industrial food systems on human diet and health according to the nature, extent, and purpose of food processing [8]. NOVA categorizes foods according to the degree of processing: in natura or minimally processed, processed culinary ingredients, processed food, and UPFs. UPFs are defined as ﻿industrial formulations manufactured from processed substances extracted or refined from whole foods. They are typically energy-dense products, with high amounts of sugar, fat, and salt, and low in dietary fiber, protein, vitamins, and minerals. UPFs also include industrial ingredients, such as hydrogenated fat, protein isolates, and additives such as colors, flavors, artificial sweeteners, and emulsifiers [9]. Some examples include products such as fast foods, cereal bars, cakes, ice cream, pizza, sausages, and soft drinks [10].

UPF intake is considered a hallmark of the Western diet and other unhealthy eating patterns such as the Prudent diet, characterized by a high intake of energy-dense and processed food, and rich in industrialized food-like products that are typically made with low-quality ingredients and deliver little nutritional value [11]. UPFs have become increasingly prevalent in the food supply system globally since they are designed to be attractive, palatable, cheap, and convenient products [12]. They account for more than 50% of the energy intake in developed countries such as the USA [13] and the UK [14] and are widely prominent in the diets of populations in lower-middle-income countries [15,16]. A recent meta-analysis of nationally representative samples showed an inverse linear relation between UPFs and less-processed foods when considered in relation to other food groups. The study also indicated that the increase in UPF intake was correlated with an increase in nutrients such as free sugars, total fats, and saturated fats, as well as a decrease in fiber, protein, potassium, zinc, and magnesium, and vitamins A, C, D, E, B3 and B12 [17]. Considering that during pregnancy women need a higher amount of the majority of nutrients to achieve optimal fetal growth and birth weight, varied diets and increased nutrient intake are needed to cope with the extra demand. Associations between maternal UPF consumption and perinatal outcomes have been investigated during the past years, however the findings are limited and inconsistent. Some studies have reported a significant association between consumption of UPF-rich diets during pregnancy and excessive gestational weight gain (GWG) [4,18], higher gestational diabetes mellitus (GDM) risk [19], hypertensive disorders of pregnancy (HDP) such as preeclampsia [20], low birth weight (LBW) [21] and preterm birth [22], while others have shown no association [7,23].

Previous systematic reviews have explored the association between maternal dietary patterns and maternal or infant outcomes [24,25,26]. However, these studies did not consider the degree of food processing, which has become an important aspect of diet quality [10].

A recent systematic review [27] reported that the highest UPF consumption negatively impacts nutrition and disease development indicators in pregnant, lactating women and children. However, a meta-analysis of the results was not conducted, and no other dietary patterns characterized by high UPF consumption were explored during the pregnancy period. 

Since the pregnancy period is considered a window of opportunity to improve dietary intake which is considered a modifiable risk factor [28], a better understanding of maternal UPF consumption effects on perinatal outcomes is crucial to promoting mother and infant health. Thus, this study aimed to determine the association between UPF-rich diet consumption by pregnant women and perinatal (maternal and neonatal) outcomes through a comprehensive systematic review with meta-analysis. The hypothesis was that a higher intake of UPF-rich diet during pregnancy is associated with adverse perinatal outcomes.

## 2. Materials and Methods

This systematic review was conducted in accordance with the Preferred Reporting Items for Systematic Reviews and Meta-Analyses (PRISMA) statement for reporting systematic reviews [29] and its protocol was registered on the International Prospective Register of Systematic Reviews (PROSPERO) under registry number CRD42021257210. The PECOS acronym (Population, Exposure, Comparison, Outcome, and Study design) was used to elaborate the guiding research question as follows: “Is consumption of a UPF-rich diet during pregnancy associated with adverse perinatal outcomes?” (Supplementary Materials Table S1).

### 2.1. Eligibility Criteria

This review included observational studies (cross-sectional, longitudinal, case-control) that reported a measure of association (relative risk, odds ratio, or β-coefficients with confidence interval) between UPF-rich diet consumption and perinatal outcomes. For this review, we considered it UPF-rich diet consumption when the evaluated food, diet, or dietary pattern included at least one food from the UPF group defined by the NOVA Food Classification System [9], such as fast foods, junk foods, processed meats, soft drinks, confectionaries, pizzas, hamburgers, candies and sweets, sweetened beverages and cookies. Diet patterns described as unhealthy dietary patterns compared to healthy patterns, and Western and Prudent diet patterns which are characterized by a higher intake of red and processed meats, beverages sweetened with sugar, sweets, desserts, industrialized food-like products, and refined grains with a high intake of energy-dense and processed foods, were also considered as a proxy for high UPF intake. No date of publication or language restriction was applied.

Studies including pregnant women with pre-existing diseases, animal studies, letters to editors, reviews, personal opinions, reviews, book chapters, editorials, congress abstracts, or any publication without primary data were excluded. Studies that evaluated individual nutrient or diet scores and studies without the required data being available even after at least two attempts to contact the authors by e-mail were also excluded. 

### 2.2. Information Sources and Search Strategy

A systematic literature search was performed on 10 June 2021, and updated on 31 May 2022, using the following databases: Medline, Embase, Scopus, Web of Science, and Lilacs (BVS). Furthermore, a gray literature search was also performed using ProQuest Dissertations and Theses Global and Google Scholar (limited to the first 200 most relevant results). The reference lists of selected articles were hand-searched to identify additional relevant publications.

The search strategy was comprised of free text words and identified terms in Medical Subject Headings and Health Sciences Descriptors for participants, exposure, and outcomes. The following terms and words combinations were searched: (pregnancy OR pregnancies OR gestation OR “pregnant women” OR “pregnant woman” OR maternal OR antenatal) AND (ultraprocessed food OR “ultra-processed food” OR “industrialized food” OR “processed food” OR “ready-to-eat meal” OR “ready-to-eat food” OR “ready-prepared food” OR “salty food” OR “high-fat diet” OR “highly processed foods” OR “refined food” OR “fast food” OR “junk food” OR “sugar-sweetened beverages” OR “soft drink” OR “unhealthy eating” OR “unhealthy diet” OR “poor diet” OR “processed meat”) AND (“perinatal outcome” OR “pregnancy outcome” OR “pregnancy complications” OR “gestational weight gain” OR “pregnancy weight gain” OR “birth outcomes” OR “birth weight” OR “neonatal weight” OR “newborn weight” OR “birth size” OR “pregnancy-induced hypertension” OR “hypertensive disorders” OR “gestational diabetes” OR “glycemic outcomes” OR “premature birth” OR “preterm birth” OR “fetal growth”). The search strategy quality was assessed by an investigator with experience in systematic reviews and expertise in the subject in accordance with the Peer Review of Electronic Search Strategies (PRESS) checklist [30]. The full search strategy for each database is available in Supplementary Materials Table S2.

### 2.3. Study Selection

The selection process for the review was independently conducted by two reviewers (WOP and ESOP) in two steps. First, the titles and abstracts of all retrieved articles were screened, according to the eligibility criteria. Then, the selected potentially eligible studies were submitted for full-text analysis. Articles that met the eligibility criteria were included in the review. Disagreements were resolved by consensus. Duplicates were identified and removed using the reference management tool Mendeley Desktop (version 1.19.8). The Rayyan QCRI software (Qatar Computing Research Institute^®^, Doha, Qatar) was used for the screening of articles.

### 2.4. Data Extraction

Data extraction was carried out by one author and cross-checking of all information was performed by a second author using a standardized spreadsheet. The following data were extracted from the original selected articles: authors and year of publication, data collection year, follow-up time, year of publication, study design, the country in which the study was conducted, sample size, age of participants, gestational age, denomination and composition of dietary components, dietary assessment methods, main outcomes, outcome measures, measures of effect size with confidence interval (CI), details of adjustment for confounding factors, and study funding/support information. When multiple estimates were reported, the results with adjustment for the highest number of confounders were used. When necessary, the respective study authors were contacted to retrieve additional information. At least two attempts were made to request missing or additional information.

### 2.5. Appraisal of Methodological Quality

Two investigators (W.O.P and E.S.O.P.) independently assessed the methodological quality of each included study using the Joanna Briggs Institute Critical Appraisal tools according to each study design (cohort, cross-sectional, and case-control) [31]. The tool consists of questions answered as “yes”, “no”, “unclear”, or “not applicable”. In this study, the risk of bias was considered low when all items were answered “yes” or “not applicable”; If the response to any item was “no” or “unclear”, a high risk of bias was expected. Disagreements were resolved by consensus. The analysis of the relative frequency of each investigated domain was presented and no scores were assigned.

### 2.6. Summary Measures and Data Analysis

The primary outcomes were the associations between UPF-rich diet consumption and maternal (GWG, GDM, or HDP) and neonatal (LBW, large for gestational age (LGA), or preterm birth) outcomes along with the respective 95% confidence intervals (CI). 

Meta-analysis was conducted when at least three studies provided data for a given outcome. In order to minimize heterogeneity, the meta-analysis included only prospective cohort studies, since it is the most adequate approach to assess associations. The overall associations were analyzed using the DerSimonian and Laird random-effects models. Based on data availability, the odds ratio (OR) and 95% CI were measured for maternal (GWG, GDM, or HDP) and neonatal (LBW, large for gestational age (LGA), or preterm birth) outcomes. If studies reported a measure of relative risk (RR), it was converted to OR using the proposed methods of Zhang and Yu [32]. Studies that report the coefficient (β) of the regression were analyzed separately. Statistical heterogeneity between studies was measured using the I-Square (I^2^). Heterogeneity was considered important if I^2^ values were higher than 40% [33]. Data analysis was performed using Stata software (StataCorp. 2019. Stata Statistical Software: Release 16.1. College Station, TX, USA: StataCorp LLC). When eligible studies did not report data in a form that could be included in the meta-analysis, they were included in the systematic review and qualitatively analyzed. Cross-sectional and case-control studies were also narratively summarized. Publication bias analyses were performed when at least ten studies were available for an outcome measure using Egger’s test with a 5% significance level and funnel plot visual inspection [33].

### 2.7. Quality of Meta-Evidence

The Grading of Recommendations Assessment, Development, and Evaluation (GRADE) system was used to evaluate the certainty of the evidence for each exposure–outcome association based on the major domains of study limitations. The quality of evidence was downgraded based on five criteria: risk of bias, inconsistency of results, indirectness of evidence, imprecision, and publication bias when it was assessed [34]. 

## 3. Results

### 3.1. Selection of Studies

**Figure 1:**
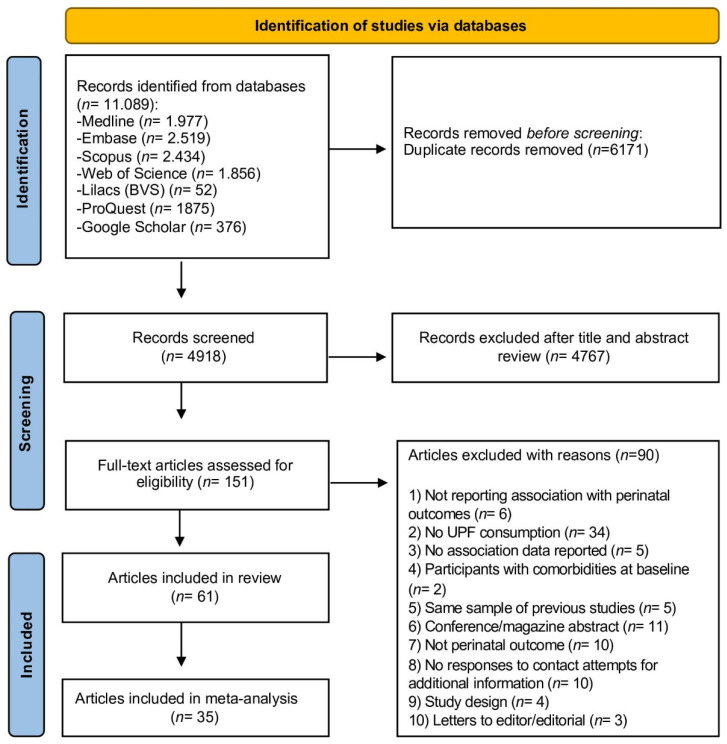
Flowchart of the study selection process. Adapted from PRISMA.

The flow chart of the study selection process is presented in Figure 1. The database search retrieved 11,089 articles. After the removal of duplicates, 4.918 article titles and abstracts were screened. Of these, 151 full-text articles were further assessed for eligibility and, finally, 61 studies [4,18,19,20,21,22,35,36,37,38,39,40,41,42,43,44,45,46,47,48,49,50,51,52,53,54,55,56,57,58,59,60,61,62,63,64,65,66,67,68,69,70,71,72,73,74,75,76,77,78,79,80,81,82,83,84,85,86,87,88,89] met the inclusion criteria and were included in this systematic review. The complete list of reasons for the exclusion of articles is presented in Supplementary Materials Table S3.

### 3.2. Study Characteristics

The articles were published between 2006 [57] and 2022 [89]. The sample ranged from 45 [4] to 94.062 [48] with 698.803 pregnant women evaluated in total. The included studies were conducted in Africa [50,51], Asia [19,35,36,37,38,39,40,41,42,43,44,45,46,47,48,49], America [4,18,52,53,54,55,56,57,58,59,60,61,62,63,64,65,89], Europe [20,21,66,67,68,69,70,71,72,73,74,75,76,77,78,79,80,81,82,83,84,85,86] and Oceania [22,87,88]. Forty-seven of the studies had a cohort design [4,18,19,20,36,40,42,44,46,48,49,50,51,52,53,54,55,56,57,58,59,60,62,63,66,67,68,69,70,71,72,73,74,75,76,77,78,79,81,82,83,84,85,86,87,88], nine were cross-sectional [22,43,45,47,61,64,65,80,89] and five case-control [21,35,37,38,41]. Maternal mean age ranged from 24 ± 8 [37] a 37 ± 4 years old [67] and gestational week from ≤6 [19] to 37 [64] in baseline. 

Regarding the exposure to UPF-rich diet consumption, seventeen articles assessed Western Diet Pattern (characterized by the presence of unhealthy foods such as savory and sweet snacks, cakes, cookies, desserts, refined grains, processed meats, fast foods, confectionaries and soft drinks) [20,35,36,37,38,39,40,41,51,57,62,67,68,71,80,83,85]; the intake of sweetened beverages was explored in twelve articles [46,49,52,56,64,70,72,73,75,78,79,82]; and specific manufactured food groups including UPF were analyzed in twelve articles [4,18,22,43,44,55,59,60,76,81,89]. In addition, studies also reported maternal consumption of junk foods [50,87], processed meats [65,69], snacks [61,84], industrial sweets [21,58,65], fast foods [19,42,50,54,66,74,77], “unhealthy food pattern” [45,86,88], “high salt pattern” [35], and ready-to-eat food [48].

Regarding to maternal outcomes, GWG was investigated in thirteen articles [4,18,36,42,51,58,64,67,77,81,84,89,90]; fifteen explored the association between maternal consumption and GDM [19,38,41,42,49,56,57,61,62,64,69,71,72,74,78]; and eight reported HDP, including maternal hypertension [20,35,39,52] and preeclampsia [20,37,39,45,75,76]. Two articles explored depressive symptoms during pregnancy [46,88]. Neonatal outcomes included LBW, investigated in eleven articles [21,40,43,44,47,48,53,65,73,80,86]; LGA, investigated in eight articles [47,50,54,66,68,73,82,87]; birth length, explored in four articles [48,54,60,86]; one publication reporting body mass index (BMI)/age at birth [59]; five reporting preterm birth [22,48,55,83,85]; and offspring congenital heart defects, examined in two publications [70,79].

### 3.3. Results of Individual Studies

A summary of the characteristics and main results of each study is presented in Table 1.

Regarding the cohort studies evaluating GWG, higher odds ratios of excessive GWG were associated with snack dietary pattern (OR: 1.01; 95% CI:1.004, 1.032) [84], UPF dietary patterns such as margarine, sugar, and chips (OR: 1.45; 95% CI: 1.06, 1.99) [81], and Western dietary pattern (OR: 4.04; 95% CI: 1.07, 15.24) [36]. Gomes et al. [18] showed that each 1% increase in energy intake from UPF was associated with a mean increase of 4.17 g in weekly gestational weight (95% CI: 0.5, 7.79). Other studies also presented an increase in GWG rate associated with a UPF-rich diet consumption. Rohatgi et al. found that each one percent increase in energy intake from UPF was associated with 1.33 kg increase in total GWG (CI: 0.3, 2.4) [4]. Similarly, Maugeri et al. showed that a Western diet consumption was associated with an increase of 1217 kg in total GWG (*p* = 0.013) [67]. A UPF rich-diet was also associated with a slight increase of 0,029 kg (β: 0.029; 95% CI: 0.012, 0,049) [42] and 0,01 kg (β: 0.010; SE: 0.003; *p* = 0.004) in weekly GWG [77]. Conversely, Hirko et al. [58] observed that intake of added sugar (including soft drinks, sugary fruit-flavored drinks, candies and cookies, cakes, pies, or brownies) was associated with a slight reduction in the likelihood of excessive GWG (OR: 0.91; 95% CI: 0.84, 0.99).

Lamyian et al. [19] observed greater chances of developing GDM among pregnant women with higher consumption of fast foods (OR: 2.12; 95% CI: 1.12, 5.43). Six cohort studies also identified an association between the consumption of UPF and a higher risk of GDM [56,57,69,71,74,78]. Three studies [49,62,71] found no significant association.

A Brazilian cohort [52] identified an association between soft drink consumption and hypertension during pregnancy (RR: 1.45; 95% CI: 1.16, 1.82). Ikem et al. [20] showed that higher consumption of the Western dietary pattern increased the odds of gestational hypertension by 18% (OR: 1.18; 95% CI: 1.05, 1.33). On the other hand, Hajianfar et al. [39] observed that consumption of the Western pattern was associated with lower chances of systolic (OR: 0.13, 95% CI: 0.04, 0.42) and diastolic (OR: 0.08; 95% CI: 0.01, 0.67) hypertension. Our results present a positive association between UPF consumption and preeclampsia observed in four cohort studies [20,39,75,76].

Depressive symptoms during pregnancy were also investigated in two cohort studies. Ker et al. [46] reported that increased consumption of sugar-sweetened beverages was associated with higher depression scores (β = 0.25; 95% CI: 0.04, 0.45). Likewise, Baskin et al. [88] found a positive association between an “unhealthy” diet (characterized by the intake of UPF and unhealthy foods such as condiments, sweets and desserts, refined grains, high-energy drinks, fast foods, hot chips, high-fat dairy, fruit juice, and red meats) and increased depressive symptoms during gestation (β = 0.19; 95% CI: 0.04, 0.34).

Regarding neonatal outcomes, Hajianfar et al. [40] and Okubo et al. [44] reported that pregnant women with the highest consumption of UPF were 5.51 (95% CI: 1.82,16.66) and 5.24 (95% CI 1.1, 24.4) times more likely to have children with LBW (<2.5 kg), respectively.

A positive association between maternal UPF consumption and higher birth weight was observed in one cohort [21] whereas no association was observed in four studies [48,53,63,73]. Maternal fast food [54,66] and soft drink [82] intake were associated with LGA birth. Moreover, Grundt et al. [73] observed an inverse association between soft drink consumption and LGA risk.

Two cohorts reported higher odds of preterm birth. Martin et al. [55] and Rasmussen et al. [83] reported that UPF consumption during pregnancy increased preterm birth odds by 53% (OR: 1.53; 95% CI: 1.02, 2.30) and 30% (OR: 1.30; 95% CI: 1.13, 1.49), respectively. In opposition to these results, two cohort studies found no significant association [48,85].

Alves-Santos et al. [54] found that fast food consumption was associated with higher odds of birth length > 90th percentile (OR: 4.81; 95% CI: 1.77, 13.07). Teixeira et al. [60] observed that women who consumed more “snacks, sandwiches, sweets and soft drinks” were significantly more likely to deliver SGA (birth weight and birth length <10th percentile) babies (RR: 1.92; 95% CI: 1.08, 3.39). Mikes et al. [86] showed that higher consumption of unhealthy foods (confectionary, fried, and processed meats) was associated with lower birth length: (β = −0.10 cm; 95% CI: −0.19, −0.01). One study explored BMI-for-age z score at birth and reported a decrease of 20.41 standard deviations (SD) (95% CI: 20.79, 20.03) associated with a diet characterized by a high intake of white bread, red and processed meat, French fries, fried chicken, and vitamin C–rich drinks [59]. Finally, two studies reported a positive association between maternal soft drink intake during pregnancy and higher odds of CHD [70,79].

Selected cross-sectional studies (n = 9) examined the association between maternal UPF consumption and perinatal outcomes. No significant association was observed for excessive GWG [64,89], GDM risk [61,64], preeclampsia [45] and LGA [47]. Three studies [43,47,65] reported a positive association between the consumption of UPF and LBW, while one study [80] (n = 303) showed no significant association. A positive association was also observed for preterm birth (OR: 1.54; 95% CI: 1.10, 2.15) [22]. 

Of the five included case-control studies, one study observed that higher maternal adherence to Western diet patterns during pregnancy was associated with higher odds of GDM risk (OR: 1.68; 95% CI: 1.04, 2.72) [41]. On the other hand, Asadi et al. did not find such an association [38]. A positive association was observed between higher consumption of UPF and higher systolic blood pressure (r = 0.110, *p* < 0.05) [35], preeclampsia (OR: 5.99; 95% CI: 3.41, 10.53) [37] and LBW (OR: 2.7; 95% CI: 1.42, 5.13) [21].

### 3.4. Risk of Bias within Individual Studies

**Figure 2:**
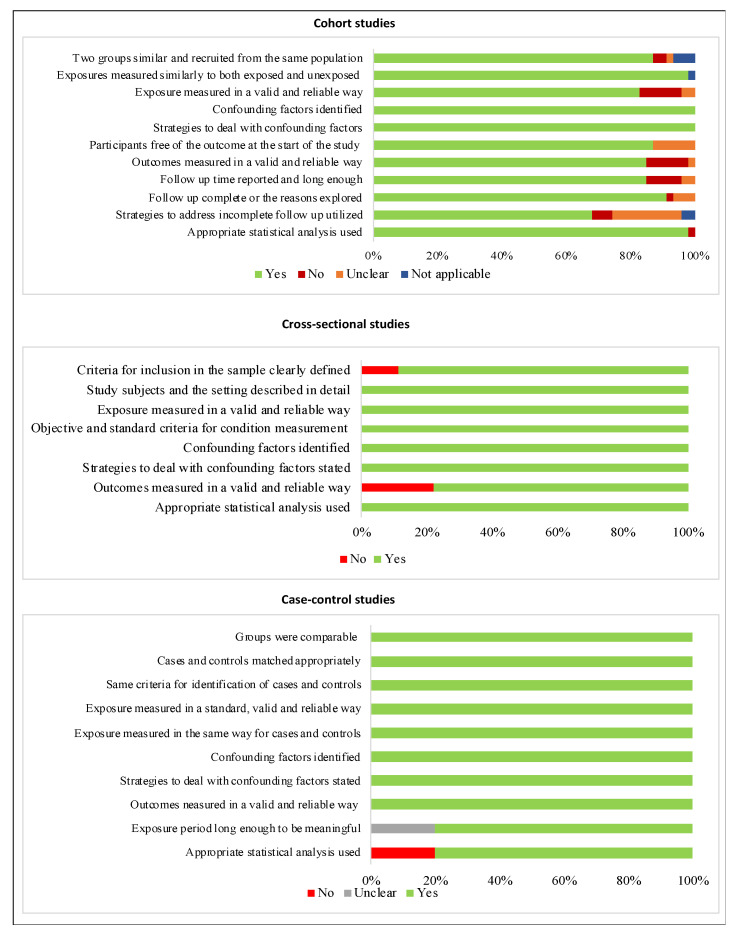
Risk of bias of the included articles according to study design.

The frequency of the items assessed as an indicator of the risk of bias in studies is illustrated according to the study design in Figure 2. Of 47 cohort studies, 24 (51%) were considered at low risk of bias [18,19,20,36,39,40,44,49,50,51,54,60,66,67,69,70,71,72,73,74,75,79,82,83]. Two indicators were accomplished in all studies: “confounding factors identified” and “strategies to deal with confounding factors stated”. Most studies were at high risk of bias due to not presenting the strategies to address incomplete follow-up, which is considered a potential source of bias [4,42,52,53,56,59,63,68,76,78,85,86,87]. Most of cross-sectional studies (77.7%) were at low risk of bias [22,43,45,61,64,65,80]. Two studies presented a high risk of bias. One article [89] did not use a reliable method to measure the assessed outcome; the other one [47] did not accomplish two of the evaluated parameters: “criteria for inclusion in the sample clearly defined” and “outcomes measured validly and reliably”. Three case-control studies (60%) were classified as having a low risk of bias [37,38,41] and two studies presented a high risk of bias due to not reporting the exposure period [21] and statistical analysis [35] clearly. The complete appraisal of the methodological quality of each article is described in Supplementary Materials (Tables S4–S6).

### 3.5. Meta-Analysis of Maternal UPF-Rich Diet Consumption and Maternal Outcomes

#### 3.5.1. Gestational Weight Gain

Five articles were pooled in the meta-analysis, including 4.576 subjects, but no association was found between maternal UPF-rich diet consumption and excessive GWG [(OR: 1.04; 95% CI: 0.92, 1.17) I^2^ = 75.22%] [36,51,58,81,84]. This association was also explored using β coefficient in five articles, including 4.384 pregnant women [4,18,42,67,77], but no significant association between UPF-rich diet consumption and GWG was found [(β = 0.02; 95% CI: −0.02, 0,06) I^2^ = 80.63%].

#### 3.5.2. Gestational Diabetes Mellitus

**Figure 3:**
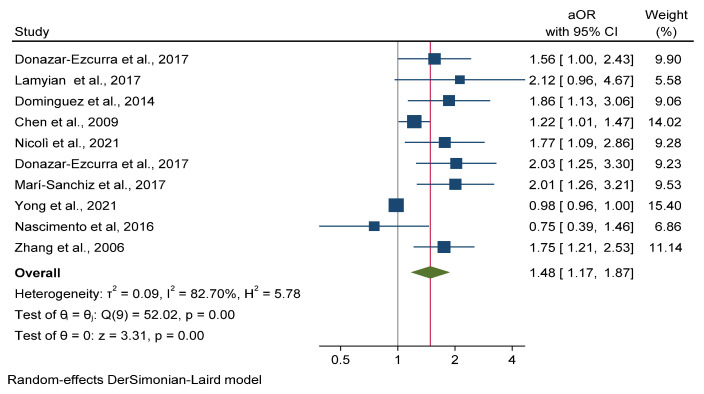
Meta-analysis of ultra-processed food rich diet *vs* gestational diabetes mellitus.

Ten cohort studies assessed the association between maternal UPF-rich diet consumption and GDM including 42.477 pregnant women [19,49,56,57,62,69,71,72,74,78]. The meta-analysis showed that higher consumption of diets rich in UPF significantly increased odds of GDM by 48% [(OR: 1.48; 95% CI: 1.17, 1.87) I^2^ = 82.70%] (Figure 3). Publication bias analysis by the funnel plot inspection (Supplementary Figure S1) showed asymmetry among the studies, which was confirmed by Egger test (*p* = 0.001). 

#### 3.5.3. Hypertensive Disorders of Pregnancy

No significant associations were observed between UPF-rich diet consumption and the odds of hypertension during pregnancy of three cohort studies, with 58.701 subjects [20,39,52] [(OR: 0.94; 95% CI: 0.52, 1.70) I^2^ = 88.80%].

**Figure 4:**
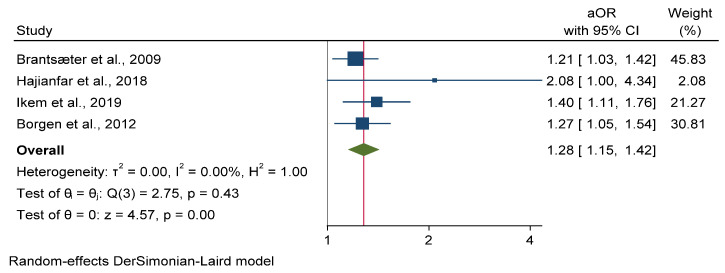
Meta-analysis of ultra-processed food rich diet vs. preeclampsia.

On the other hand, the consumption of UPF-rich diets was found to be associated with 28% higher odds of preeclampsia in four cohort studies [20,39,75,76] involving 112.307 subjects [(OR: 1.28; 95% CI: 1.15, 1.42) I^2^ = 0.00%] (Figure 4). 

### 3.6. Meta-Analysis of Maternal UPF-Rich Diet Consumption and Neonatal Outcomes

#### 3.6.1. Low Birth Weight

Five eligible cohort studies that provided an estimate of the association between maternal UPF-rich diet consumption and LBW were included in the meta-analysis [40,44,48,53,73], involving 146.617 subjects. However, no significant association was presented [(OR: 1.08; 95% CI: 0.90, 1.30) I^2^ = 74.59%].

#### 3.6.2. Large for Gestational Age

Three eligible cohort studies (n = 52.468) investigated the association between maternal UPF-rich diet consumption and LGA. [54,66,73]. Meta-analysis results revealed no significant association between UPF-rich diet consumption and odds of LGA [(OR: 2.10; 95% CI: 0.71, 6,25) I^2^ = 84.61%].

#### 3.6.3. Preterm Birth

The meta-analysis showed no significant association [(OR: 1.13; 95% CI: 0.97, 1.32) I^2^ = 76.25%] regarding the association between four cohort studies (n= 233.308) which evaluated the UPF-rich diet consumption and the odds of preterm birth. E [48,55,83,85]. 

### 3.7. Certainty of Evidence

The GRADE assessment was moderate for maternal UPF-rich diet consumption and preeclampsia (⨁⨁⨁◯) and very low (⨁◯◯◯) for GWG, GDM, LBW, LGA, and preterm birth (Table 2).

## 4. Discussion

The present systematic review highlights the role of the maternal diet, including the consequences of UPF-rich diet consumption on perinatal adverse outcomes. 

There is growing evidence that high consumption of UPFs is indicative of low diet quality and associated with a higher risk of coronary heart disease, cancer, cerebrovascular and metabolic diseases, hypertension, worse cardiometabolic risk profile, and a higher risk of all-cause mortality in adult and older populations [91,92,93]. Regarding the pregnancy period, a recent systematic review [27] indicated that high UPF consumption in pregnancy, lactation, and infancy had negative repercussions on health in general but no meta-analysis was performed. To our knowledge, this is the first study with meta-analysis to assess the effect of UPF-rich diet consumption, through unhealthy dietary patterns, Western foods and UPF intake, by pregnant women and perinatal outcomes, and is the most up-to-date and comprehensive systematic review on this topic.

The significant association found between higher maternal consumption of UPF-rich diets and higher risk of GDM is corroborated by previous studies. A meta-analysis of cohort studies showed that the Western dietary pattern, determined by high intakes of red and processed meat, fried foods, and refined grains, could increase the risk of GDM [94]. Quan et al. also showed that consumption of fast food had a positive association with higher GDM risk [95]. Furthermore, diets presenting high amount of UPFs are frequently rich in sugars and refined grains products, recognized risk factors for GDM [15], endorsing the results of this meta-analysis. In contrast to our results, Kibret et al. [96] found no association between the Western diet pattern and GDM, which may be due to the inclusion of studies assessing UPF-rich dietary patterns as well as soft drinks intake and processed meats alone in the present GDM meta-analysis.

Another interesting finding was a significant association between UPF-rich diets consumption and preeclampsia. A previous recent study with meta-analysis investigated the effects of maternal dietary patterns on pregnancy and reported that maternal adherence to an unhealthy diet was associated with 23% higher odds of HDP, including preeclampsia [97]. Another study also found a significant association between higher adherence to a Western dietary pattern, an unhealthy diet pattern characterized by a high amount of UPF such as processed meat, soft drinks, and refined foods, and increased risk of preeclampsia [98], corroborating our results. 

Although the causes of preeclampsia are multifactorial, some risk factors are associated with the development of HDP, such as women experiencing their first pregnancy, twin pregnancy, chronic hypertension, GDM, maternal obesity, and maternal age over 35 years. In addition, healthy lifestyle habits before and during pregnancy can influence the severity of the outcomes [99]. UPFs are rich in sodium, free or added sugars, saturated and trans fats, high energy density, and low in fiber, potassium, and micronutrients [15]. In this context, maternal diet quality has clinical significance given the established association of preeclampsia with maternal and fetal complications such as maternal mortality, perinatal deaths, preterm birth, and intrauterine growth restriction. Moreover, pregnant women affected by HDP have a higher risk of cardiovascular disease in later life, regardless of other risk factors [100,101].

Despite the lack of significant association between UPF-rich diets consumption and excessive GWG, evidence indicates that GWG is significantly correlated with maternal energy intake [102,103,104]. A recent systematic review reported that dietary patterns with ultra-processed components rich in fat and sugars presented an association with higher GWG [89]. Sartorelli et al. [23] also showed that women classified into the highest tertile of UPFs intake had a three times higher chance of obesity when compared to women with the lowest intake of these foods. Thus, monitoring this trend in pregnant women should be an important healthcare concern objective since excessive GWG is associated with greater chances of hypertensive disorders, cesarean delivery, and LGA newborns [105,106,107], and a strong predictor of postpartum weight retention, contributing to obesity in later life [108,109].

The development of GDM and preeclampsia could be related to the low nutritional quality of the UFP-rich diet. The low quality of carbohydrates found in UPFs may impair glycemic control [110], especially from the second trimester when anti-insulin hormones, such as estrogens, progesterone, and chorionic somatomammotropin, act by decreasing the power of insulin action, making more glucose available in the bloodstream [111]. The risk of pregnancy complications such as preeclampsia has been linked with maternal oxidative stress in the middle of pregnancy [112]. The findings of a multicenter study showed that oxidative stress could be reduced by sufficient intakes of fruit, vegetables, and vitamin C [113], and Pistollato et al. (2015) reported a lower likelihood of pregnancy-induced hypertension or preeclampsia when the diet pattern comprised intake of plant-derived foodstuffs and vegetables [114]. Thus, higher UPFs intake may impact and reduce consumption of antioxidants and foment oxidative stress status during pregnancy. 

Regarding neonatal outcomes, the present meta-analysis showed no association between maternal UPF-rich diet consumption and neonatal birth outcomes such as birth weight and preterm birth. Endorsing our results, a study with a meta-analysis conducted by Abdollahi et al. [97] showed no association between an unhealthy pattern and birth weight. Kibret et al. [96] also found that a dietary pattern rich in UPF, a Western dietary pattern, did not increase the odds of preterm birth, corroborating our findings. 

Nonetheless, the importance of maternal diet in early pregnancy for neonatal health is well documented. Birth weight is an important parameter for assessing newborn health conditions and development, and also is used as one of the basic indicators in the global reference list of the World Health Organization (WHO) [115]. In a meta-analysis conducted with observational studies, Chia et al. [26] reported that unhealthy dietary patterns, characterized by high intakes of refined grains, processed meat, and foods high in saturated fat or sugar, were associated with lower birth weight and a trend towards a higher risk of preterm birth. The study of Rohatgi et al. [4] reported that higher maternal UPF consumption was associated with increased adiposity in the neonate. Taken together, the evidence suggests that maternal diet quality, including UPF consumption, might affect neonatal health. 

The etiology of preterm birth is still not well understood, and most cases do not have clear determinants. Some studies reported greater chances of preterm birth observed in pregnant women with high consumption of highly processed foods high in fat and sugar, while the consumption of a healthy diet, rich in fruits, vegetables, and whole grains, appeared to significantly reduce the risk [22,55,83]. Moreover, a meta-analysis of nine cohort studies indicated that higher adherence to a healthy dietary pattern significantly decreased the odds of preterm birth [96].

The results of the present study indicate important public health implications, since higher UPF consumption may worsen perinatal health outcomes. The positive association between UPF-rich diet consumption and GDM and preeclampsia suggests that the consumption of diets rich in UPFs, such as those with high factor loadings for fast foods, junk foods, processed meats, soft drinks, pizzas, hamburgers, candies and sweets, should be discouraged during pregnancy whereas increasing the proportion of in natura and minimally processed food in the diet should be reinforced. Furthermore, prioritizing a healthy lifestyle, which considers adequate food intake, regular physical exercise, regular sleep, and adequate gestational weight gain is mandatory for this population group. This study provides insights to guide policies on pregnancy healthcare as well as nutritional interventions in prenatal services. Further studies with robust methodological quality, such as larger samples and using a more accurate dietary assessment instrument, are needed to clarify the findings on this topic.

The NOVA food categorization classifies foods and beverages “according to the extent and purpose of industrial processing” and defines UPF as “formulations of ingredients, most of exclusive industrial use, that result from a series of industrial processes” (hence “ultra-processed”) [10]. Considering that unhealthy dietary patterns, such as Western and Prudent diets, are characterized by a high consumption of UPF, we speculate that our results provide an effort to measure the UPF consumption association with perinatal outcomes, since diet is a modifiable risk factor. This study has several strengths. To date, this is the first study conducted with a meta-analysis on the topic. A comprehensive search strategy was carried out using a robust and appropriate methodology according to Cochrane Handbook and PRISMA guidelines. Moreover, many subjects were included for each pooled outcome, increasing the generalizability of the results. In addition, the methodological quality of the included studies was assessed independently, and the GRADE system was used to assess the certainty of the evidence of each exposure–outcome association. Despite the few studies in the pregnancy group specifically evaluating UPFs intake, out of the 61 studies included in the review, 83% found a significant association between UPF-rich diets consumption and adverse health outcomes. These data demonstrate the important impact on public health in the maternal and child group and may support future nutritional recommendations for these populations.

Some limitations are also noteworthy. First, the study did not exclusively evaluate UPF consumption, but we speculate that unhealthy and Western dietary patterns may be considered as a proxy for UPF intake. Second, applied dietary assessments of the included studies were not specifically designed for the NOVA classification system. Third, high heterogeneity between studies was observed in many analyses considering the nature of the observational nutritional studies. This is expected because of the diverse characteristics of subjects, the different dietary approaches, and the variance between outcome assessment methods. Fourth, the lack of significant results in perinatal outcomes may be due to the small number of included articles for each outcome, thus it was not possible to perform subgroups analysis to seek the source of heterogeneity. Lastly, publication bias was observed, so, studies that had negative results might not have been submitted for publication and were not included. 

Finally, maternal nutrition for successful pregnancy outcomes cannot be addressed during pregnancy alone. A varied diet rich in protein sources, fruit, and vegetables should be consumed by women who intend to become pregnant and during pregnancy as a component of prenatal care. The results presented here suggest that nutritional recommendations should focus not only on foods and nutrients amounts but also on the degree of food processing. 

## 5. Conclusions

This study indicates a positive association between maternal UPF-rich diet consumption during pregnancy and increased risk of developing gestational diabetes mellitus and preeclampsia. These findings corroborate the adverse effects of consumption of diets rich in UPF during pregnancy and highlight the need to monitor and reduce UPF-rich diet consumption specifically during the gestational period, as a strategy to prevent adverse perinatal outcomes. 

## Figures and Tables

**Table 1:** Summary of included studies characteristics.

Author, Year Country	Study Design	Age (Years)	GW (Range or Mean)	Sample n =	Exposure	Outcome	Main Results
Abbasi et al., 2019 Iran [37]	Case-control	case: 24 ± 8 control: 26 ± 6	>20 weeks	case: 170 control: 340	WDP (red and processed meat, fried potatoes, pickles, sweets, pizza)	Risk of preeclampsia	The Western dietary pattern associated with preeclampsia: OR: 5.99; 95% CI: 3.414, 10.53; *p* < 0.001)
Alves-Santos et al., 2019 Brazil [54]	Prospective Cohort	26.7 ± 5.5	5–13 weeks	193	Fast foods and candies (fast food and snacks; cakes, cookies, or crackers; and candies or desserts)	LGA Birth Length (BL)	Fast food and candies dietary pattern associated with LGA newborn: OR: 4.38; 95% CI: 1.32, 14.48 Fast food and candies dietary pattern associated with the newborn with BL > 90th percentile: OR: 4.81; 95% CI: 1.77, 13.07
Amezcua-Prieto et al., 2019 Spain [21]	Case-control	NR	NR	518	Industrial sweets	SGA	Intake of industrial sweets associated with odds of having an SGA newborn (OR: 2.70; 95% CI: 1.42, 5.13).
Ancira-Moreno et al., 2020 Mexico [53]	Prospective Cohort	25.08 ± 5.8	2nd and 3rd trimester	660	Mixed dietary patterns (sugary drinks, juices and sodas, red and processed meat, cereals)	LBW	The mixed dietary pattern associated risk LBW infant: (OR: 1.58; 95% CI: 0.63, 3.44)
Angali, Shahri, Borazjani, 2020, Iran [42]	Prospective Cohort	≥18 years	<13 weeks	488	“High fat - fast food” pattern (refined cereal, processed meat and high-fat dairy and juices)	GWG and hyperglycemia	High fat-fast food patterns associated with higher GWG (β: 0.029; 95% CI: 0.012, 0.049).
Asadi et al., 2019 Iran [38]	Case-control	case: 29 ± 5.17 control: 27.5 ± 4.92	24–28 weeks	case: 130 control: 148	WDP (SSB, refined grain products, fast foods, salty snacks, sweets and biscuit, mayonnaise)	GDM	The prudent dietary pattern associated with GDM risk: (OR: 0.88; 95% CI: 0.44, 0.99)
Barbosa et al., 2021 Brazil [52]	Prospective Cohort	>14	22–25 weeks	2750	Soft drinks	Gestational Hypertension (GH)	Soft drink consumption > 7 times per week associated with GH: (RR: 1.45; 95% CI: 1.16, 1.82; *p* = 0.001)
Bärebring et al., 2016 Sweden [84]	Prospective Cohort	32.1 (IQR: 30.8–35.3)	35.9 weeks (IQR: 35.1–36.4)	95	Snacks pattern (sweets, cakes, biscuits, potato chips, popcorn)	GWG	Snacks pattern associated with excessive GWG (OR: 1.018; 95% CI: 1.004, 1.032).
Baskin et al., 2015 Australia [88]	Prospective Cohort	30.55 ± 4.24	16 weeks	167	Unhealthy dietary patterns (sweets and desserts, refined grains, high- energy drinks, fast foods, hot chips, high-fat dairy, fruit juice and red meats)	Depressive symptoms	An unhealthy diet at T2 is associated with depressive symptoms: β: 0.19; 95% CI=0.04, 0.34; *p* < 0.05
Borgen et al., 2012 Norway [75]	Prospective Cohort	>18 years	15 weeks	32,933	SSB	Preeclampsia	Sugar-sweetened beverages associated with increased risk of preeclampsia: OR: 1.27; 95% CI: 1.05, 1.54
Brantsæter et al., 2009 Norway [76]	Prospective Cohort	>18	20.7 weeks (SD ± 3.7)	23,423	Dietary patterns (Processed meat products, white bread, French fries, salty snacks, and sugar-sweetened drinks)	Risk of preeclampsia	Processed food patterns are associated with increased risk of developing preeclampsia (OR: 1.21; 95% CI: 1.03, 1.42).
Chen et al., 2009 USA [56]	Prospective Cohort	24–44	NR	13,475	SSB	Risk of gestational diabetes mellitus (GDM)	Intake of sugar-sweetened cola associated with risk of GDM (RR: 1.22; 95% Cl: 1.01, 1.47).
Chen et al., 2020 China [35]	Case-control	case: 28 ± 1.3 control: 28 ± 1.5	>22 weeks	case: 1290 control: 1290	High-salt pattern (pickled vegetables, processed and cooked meat, fish and shrimp, bacon and salted fish, bean sauce)	Hypertensive disorder during pregnancy	High-salt pattern diets associated with higher systolic blood pressure: (r: 0.110; *p* < 0.05)
Coelho et al., 2015 Brazil [63]	Prospective Cohort	24.7 ± 6.1	≥22 weeks	1298	Snack dietary patterns (sandwich cookies, salty snacks, chocolate, and chocolate drink)	Birth weight	Snack dietary patterns positively associated with birth weight: (β: 56.64; *p* = 0.04) in pregnant adolescents.
Dale et al., 2019 Norway [79]	Prospective Cohort	≥18	16-18 weeks	88,514	SSB	CHD	25–70 mL/day sucrose-sweetened soft beverages associated with non-severe CHD (RR:1.30; 95% CI: 1.07, 1.58) and (RR: 1.27; 95% CI: 1.06, 1.52) for ≥70 mL/day.
Dominguez et al., 2014 Spain [74]	Prospective Cohort	>18	NR	3048	Fast food	GDM	Fast food consumption associated with GDM risk: (OR: 1.86; 95% CI: 1.13, 3.06)
Donazar-Ezcurra et al., 2017 Spain [71]	Prospective Cohort	>18	NR	3455	WDP (red meat, high-fat processed meats, potatoes, commercial bakery products, whole dairy products, fast foods, sauces, pre-cooked foods, eggs, soft drinks and sweets, chocolates)	GDM	The Western dietary pattern associated with GDM incidence: (OR: 1.56; 95% CI: 1.00, 2.43; *p* = 0.05)
Donazar-Ezcurra et al., 2017 Spain [72]	Prospective Cohort	>18	NR	3396	Soft drinks	GDM	Sugar-sweetened soft drinks (SSSD) associated with GDM: (OR: 2.06; 95% CI: 1.28, 3.34; *p*: 0.004)
Englund-Ögge et al., 2014 Norway [85]	Prospective Cohort	<20 to ≥40	15 weeks	66,000	WDP (salty snacks, chocolates and sweets, French fries, cakes, white bread, ketchup, dairy desserts, SSB, mayonnaise, processed meat, waffles, pancakes, cookies)	Preterm delivery	Western diet pattern associated with risk of preterm delivery (Hazard Ratio: 1.02; 95% CI: 0.92, 1.13).
Englund-Ögge et al., 2019 Norway [68]	Prospective Cohort	>18 years	15 weeks	65,904	WDP (salty snacks, chocolate and sweets, cakes, French fries, white bread, ketchup, SSB, processed meat products, and pasta)	LGA	The prudent pattern associated with decreased LGA risk: (OR: 0.84; 95% CI: 0.75, 0.94) The traditional group associated with increased LGA risk: (OR: 1.12; 95% CI: 1.02, 1.24)
Ferreira et al., 2022 Brazil [89]	Cross-sectional	28 (IQR 19–45)	NR	260	Dietary patterns (sweets, snacks and cookies)	GWG	Women with greater adherence to “Pattern 2” (sweets, snacks, and cookies) during pregnancy were less likely to have inadequate GWG (OR: 0.14; 95% CI = 0.03, 0.60)
Garay et al., 2019 United Kingdom [80]	Cross-sectional	18–45 years	NR	303	WDP (cakes/biscuits/ice cream, chips/crisps, processed meat, takeout, chocolate, soft drinks)	CBWC	Health-conscious dietary pattern associated with increased CBWC (OR: 4.75; 95% CI: 1.17, 8.33; *p* = 0.010) “Western Diet” associated with increased CBWC (β: −2.64; 95% CI: −5.87, 0.59; *p* = 0.109)
Gomes et al., 2020 Brazil [18]	Prospective Cohort	≥18 years	All trimesters	259	UPF energy (cookies, sweets, SSB, reconstituted meats, crackers, packaged chips, frozen dinners, ultra-processed breads)	GWG	Energy percentage derived from UPF associated with average weekly GWG (β: 4.17; 95% CI 0.55, 7.79).
Grieger, et al., 2014 Australia [22]	Cross-sectional	>18	13 weeks	309	Dietary patterns (high-fat/sugar/takeaway: takeaway foods, potato chips, refined grains, and added sugar)	Preterm delivery	High-fat/sugar/takeaway pattern associated with preterm birth: (OR: 1.54; 95% CI: 1.10, 2.15; *p* = 0.011)
Grundt et al., 2016 Norway [73]	Prospective Cohort	>18	15 weeks	50,280	SSC	BW	Each 100 mL intake of SSC associated with: 7.8 g decrease in BW (95% CI: −10.3, 5.3); decreased risk of BW > 4.5 kg (OR: 0.94; 95% CI: 0.90, 0.97) and increased risk of BW < 2.5 kg (OR: 1.05; 95% CI: 0.99, 1.10).
Günther et al., 2019 Germany [66]	Prospective Cohort	30.3 ± 4.4	<12 weeks	1995	Fast foods	LGA	Fast food consumption associated with LGA: (OR 3.14; 95% CI: 1.26,7.84; *p* = 0.014)
Hajianfar et al., 2018 Iran [39]	Prospective Cohort	20–40	8–16 weeks	812	WDP (processed meats, fruits juice, citrus, nuts, desserts and sweets, potato, legumes, coffee, egg, pizza, high fat dairy, and soft drinks)	Preeclampsia Hypertension	The Western dietary pattern is associated with: Preeclampsia: (OR: 2.08; 95% CI: 1,4.36, *p* = 0.02) High systolic blood pressure: (OR: 0.13; 95% CI: 0.04, 0.42; *p* = 0.002)
Hajianfar et al., 2018 Iran [40]	Prospective Cohort	29.4 ± 4.85	8–16 weeks	812	WDP (processed meats, fruits juice, citrus, nuts, desserts and sweets, potato, legumes, coffee, egg, pizza, high fat dairy, and soft drinks)	LBW	Western dietary pattern (top quartile) associated with LBW infant: (OR: 5.51; 95% CI: 1.82, 16.66; *p* = 0.001)
Hirko et al., 2020 USA [58]	Prospective Cohort	mean: 27	mean: 13.4 weeks	327	Dietary patterns (added sugar: soda, fruit-flavored drinks with sugar, pastries—donuts, sweet rolls, Danish, and cookies, cake, pie, or brownies)	GWG	Higher added sugar intake associated with excessive GWG (OR: 0.91; 95% CI: 0.84, 0.99)
Ikem et al., 2019 Denmark [20]	Prospective Cohort	25–30	12 weeks	55,139	WDP (potatoes, French fries, bread white, pork, beef veal, meat mixed, meat cold and dressing sauce)	Gestational hypertension Preeclampsia	Western diet associated with GH: (OR: 1.18; 95% CI: 1.05, 1.33) Preeclampsia: (OR: 1.40; 95% CI: 1.11, 1.76):
Itani et al., 2020 United Arab Emirates [36]	Prospective Cohort	19–40	27–42 weeks	242	WDP (sweets, sweetened beverages, added sugars, fast food, eggs, and offal)	GWG	The Western pattern is associated with excessive gestational weight gain (OR: 4.04; 95% CI: 1.07, 15.24)The western pattern is associated with gestational weight gain rate (OR: 4.38; 95% CI: 1.28, 15.03)
Ker et al., 2021 Taiwan [46]	Prospective Cohort	33.9 ± 4.6	All trimesters	196	SSB	Postpartum depression	SSB intake associated with increased EPDS scores: (β: 0.25; 95% CI: 0.04, 0.45) during the first and second trimesters
Lamyian et al., 2017 Iran [19]	Prospective Cohort	18–45 years	≤6 weeks	1026	Fast food	GDM	Fast food consumption (≥175 g/week) associated with GDM risk: (OR: 2.12; 95% CI: 1.12, 5.43; *p*-trend: 0.03
Liu et al., 2021 China [47]	Cross-sectional	26.88 ± 4.62	All trimesters	7934	Dietary patterns (snacks pattern: beverages, sweetmeat, fast-food, dairy and eggs)	Macrossomia SGA	Snacks pattern associated with: risk of macrosomia: (OR: 1.265; 95% CI: 1.000, 1.602) SGA: (OR: 1.260; 95% CI: 1.056, 1.505).
Loy, Marhazlina; Jan 2013 Malaysia [43]	Cross-sectional	29.7 ± 4.8	33.66 ± 3.95 weeks	108	Dietary patterns (confectioneries: cake, cookies, chocolate, candy, sweetened condensed milk)	LBW	Confectioneries food intake associated with lower birth weight: (β: −1.999; *p* = 0.013)
Marí-Sanchiz et al., 2017 Spain [69]	Prospective Cohort	>18	NR	3298	UPF (Processed meat)	GDM	Processed meat consumption associated with GDM: (OR: 2.01; 95% CI: 1.26, 3.21; *p*-trend 0.003)
Marquez, 2012 USA [64]	Cross-sectional	18–49	≥37 weeks	290	SSB	GWG	A high intake of regular soda is associated with an increased risk of Excessive GWG (OR: 1.41; 95% CI: 0.60, 3.31).
Martin et al., 2016 Sweden [59]	Prospective Cohort	16–47	39 ± 2 weeks	389	Dietary patterns (latent class 3: white bread, red and processed meats, fried chicken, French fries, and vitamin C–rich drinks)	BMI-for-age at birth	Association between the latent class 3 diet (processed food) and BMI-for-age z-score at birth:(β: −0.41; 95% CI: −0.79, −0.03).
Martin et al., 2015 USA [55]	Prospective Cohort	NR	24–29 weeks	3941	Dietary patterns (hamburgers or cheeseburgers, white potatoes, fried chicken, beans, corn, spaghetti dishes, cheese dishes, processed meats, biscuits, and ice cream)	Preterm birth	Diet characterized by ultra-processed food associated with preterm birth: (OR: 1.53; 95% CI: 1.02, 2.30)
Maugeri et al., 2019 Italy [67]	Prospective Cohort	15–50 (Mean: 37)	4–20 weeks (Mean: 16)	232	WDP (high intake of red meat, fries, dipping sauces, salty snacks and alcoholic drinks)	GWG	Western dietary patterns associated with GWG: (β: 1.217; Standard Error: 0.487; *p* = 0.013)
Mikeš et al., 2021 Czech Republic [86]	Prospective Cohort	25 ± 5	32 weeks	4320	Unhealthy Dietary pattern: (pizza, fish products, processed meat, sausages, smoked meat, hamburgers, and confectionary foods, sugary drinks, cakes, chocolate and sweets).	Birth Weight Birth Length	A 1-unit increase in the unhealthy pattern score was associated with a mean birth weight reduction of −23.8 g (95% CI: −44.4, −3.3; *p* = 0.023); a mean birth length reduction of −0.10 cm (95% CI: −0.19, −0.01; *p* = 0.040).
Mitku et al., 2020 South Africa [50]	Prospective Cohort	<25 to >30	1st and 2nd trimesters	687	Junk food (sweets, muffins, chips, mixed salad, fruit juice, fizzy soft drinks, vetkoek, coffee creamer, cooking oil, hamburgers, cooked vegetables, cereals rice, margarine)	Birth Weight	Junk food intake is associated with an increase in birth weight (*p* < 0.001).
Nascimento et al., 2016 Brazil [62]	Prospective Cohort	26.2 ± 5.8	26.4 weeks (SD ± 0.8)	841	WDP (white bread, savory, sweet, chocolate, cookies, soft drinks, pasta, fried food, pizza, chicken, canned food)	GDM	Association between GDM incidence and dietary patterns (RR: 0.78; 95%CI: 0.43, 1.43)
Nicolì et al., 2021 Italy [78]	Prospective Cohort	35.75 ± 5.53	NR	376	Soft drink	GDM	Non-nutritive-sweetened soft drink consumption associated with GDM (OR: 1.766; 95% CI: 1.089, 2.863; *p* = 0.021)
Okubo et al., 2012 Japan [44]	Prospective Cohort	≥18	All trimesters	803	Dietary patterns (wheat products pattern: bread, confectioneries, fruit and vegetable juice, and soft drinks)	SGA birth	Wheat products pattern associated with SGA infant: (OR: 5.2; 95% CI: 1.1, 24.4)
Rasmussen et al., 2014 Denmark [83]	Prospective Cohort	21–39	2nd trimester	69,305	WDP (French fries, white bread, meat mixed, margarine, dressing sauce, chocolate milk, soft drink, cakes, chocolate, candy, sweet spread, dessert dairy)	Preterm Birth	Western diet associated with preterm delivery (OR: 1.30; 95% CI: 1.13, 1.49)
Rodrigues, Azeredo, Silva, 2020, Brazil [65]	Cross-sectional	24.9 ± 6.5	39.4 weeks (SD ± 1.2)	99	Processed meat	LBW	Maternal consumption of sausages associated with LBW: (OR: 1.46; 95% CI: 1.02, 2.10)
Rohatgi et al., 2017 USA [4]	Prospective Cohort	27.2 ± 5.1	32–37 weeks	45	UPF energy intake	GWG	Each 1% increase in UPF energy intake associated with increase in GWG: (β: 1.33; 95% CI: 0.3, 2.4; *p* = 0.016)
Schmidt et al., 2020 Denmark [70]	Prospective Cohort	NR	12 weeks	66,387	Soft drinks	CHD	High intake of sugar-sweetened carbonated beverages (≥4 servings) associated with CHD: (OR: 2.41; 95% CI: 1.26, 4.64; *p*-trend = 0.03.)
Sedaghat et al., 2017 Iran [41]	Case-control	case: 29.64 ± 4.52 control: 29.76 ± 4.26	case: 29.39 ± 4.74 weeks control: 31.19 ± 3.53 weeks	case: 122 control: 266	WDP (sweet snacks, mayonnaise, SSB, salty snacks, solid fats, high-fat dairy, red and processed meat, and tea and coffee)	GDM	Western dietary patterns associated with GDM risk: (OR: 1.68; 95% CI: 1.04, 2.27)
Tamada et al., 2021 Japan [48]	Prospective Cohort	30.7 years (SD ± 5.1)	14.4 weeks (SD ± 5.6)	94,062	Ready-made meals (pre-packed foods, instant noodles, soup)	Stillbirth Preterm Birth LBW	Ready-made meals associated with stillbirth: (OR: 2.632; 95% CI: 1.507, 4.597; q = 0.007); Preterm birth: (OR: 0.993; 95% CI: 0.887, 1.125) LBW: (OR: 0.961; 95% CI: 0.875, 10.56)
Teixeira et al., 2020 Brazil [60]	Prospective Cohort	mean: 25.9	10–11 weeks	299	Dietary patterns (processed meats, sandwiches and snacks, sandwich sauces, desserts and sweets, soft drinks)	SGA	Dietary pattern with snacks, sandwiches, sweets, and soft drinks associated with the risk to deliver SGA babies: (RR: 1.92; 95% CI: 1.08, 3.39)
Tielemans et al., 2015 Netherlands [81]	Prospective Cohort	31.6 (IQR ± 4.3)	13.4 weeks (IQR: 12.2–15.5)	3374	Dietary patterns (margarine—solid and liquid, sugar and confectionary, cakes, chocolate, candy, snacks)	GWG	Margarine, sugar, and snacks pattern are associated with a higher prevalence of excessive GWG: (OR: 1.45; 95% CI: 1.06, 1.99)
Uusitalo et al., 2009 Finland [77]	Prospective Cohort	29.2 ± 5.2	10 weeks	3360	Dietary patterns (fast food: sweets, fast food, snacks, chocolate, fried potatoes, soft drinks, high-fat pastry, cream, fruit juices, white bread, processed meat, sausage)	GWG	Fast food patterns associated with weight gain rate: (β: 0.010; SE: 0.003; *p* = 0.004)
Wen et al., 2013 Australia [87]	Prospective Cohort	>16	24–34 weeks	368	Junk food diet (soft drinks, processed meat, meals, chips or French fries)	LGA	Junk food diet versus without a junk food diet associated with a newborn LGA: (OR: 0.36; 95% CI: 0.14, 0.91; *p* = 0.03)
Wrottesley, Pisa & Norris, 2017; South Africa [51]	Prospective Cohort	≥18	All trimesters	538	WDP (white bread, cheese and cottage cheese, red meat, processed meat, roast potatoes and chips, sweets, chocolate, soft drinks, miscellaneous)	GWG	Western dietary pattern associated with excessive GWG (OR: 1.07; 95% CI: 0.78, 1.45; *p* = 0.682)
Yong et al., 2021 Malaysia [49]	Prospective Cohort	30.01 ±4.48	1st trimester	452	Beverages (carbonated and juices)	GDM	Higher fruit juice intake associated with GDM (OR: 0.92; 95% CI: 0.89, 0.98).
Zareei et al., 2019 Iran [45]	Cross-sectional	28.96 ± 5.85	NR	82	Dietary patterns (unhealthy dietary patterns: mayonnaise, fries, red meat, soft drinks, pizza, snacks, sweets and dessert, refined cereal, hydrogenated oils, high-fat dairy products, sugar, processed meat, broth.)	Preeclampsia	The unhealthy dietary pattern associated with preeclampsia (OR: 1.381; 95% CI: 0.462, 4.126, *p* = 0.564)
Zhang et al., 2006 USA [57]	Prospective Cohort	>18	NR	13,110	WDP (red and processed meat, refined grain products, sweets, French fries and pizza)	GDM	Western pattern score associated with GDM risk (RR: 1.63; 95% CI: 1.20, 2.21; *p* = 0.001); Red meat associated with GDM risk: (RR: 1.61; 95% CI: 1.25, 2.07) Processed meat associated with GDM risk: (RR: 1.64; 95% CI: 1.13, 2.38)
Zhu et al., 2017 Denmark [82]	Prospective Cohort	>18	25 weeks	918	Soft drinks	Birth weight	Daily soft drinks consumption associated with offspring risk of LGA: (RR: 1.57; 95% CI: 1.05, 2.35)
Zuccolotto et al., 2019 Brazil [61]	Cross-sectional	27.6 ± 5.4	24–39 weeks	785	Snack dietary patterns (breads; butter and margarine; Processed meat, sweets, chocolate milk and cappuccino)	GDM	Dietary patterns associated with GDM risk: (OR: 1.01; 95% CI: 0.63, 1.63)

BMI: body mass index; BW: birth weight; CBWC: customized birthweight centiles; CI: confidence interval; CHD: congenital heart defects; EPDS: Edinburgh Postpartum Depression Scores; GDM: gestational diabetes mellitus; GWG: gestational weight gain; IQR: interquartile range; LBW: low birth weight; LGA: large for gestational age; NR: not reported; OR: odds ratio; RR: relative risk; SD: standard deviation; SGA: small for gestational age; SSB: sugar-sweetened Beverage; SSC: sugar-sweetened carbonated beverages; UPF: ultra-processed food; WDP: Western dietary pattern.

**Table 2:** GRADE evidence profile for maternal UPF consumption and perinatal outcomes.

Outcomes	Studies (n, References)	Risk of Bias	Inconsistency ^a^	Indirectness ^b^	Imprecision ^c^	Publication Bias	Certainty
Maternal Outcomes							
Excessive Gestational Weight Gain	5 [36,51,58,81,84]	Not serious	Serious	Not serious	Not serious	Not assessed ^d^	⨁◯◯◯ Very low
Gestational Weight Gain	5 [4,18,42,67,77]	Not serious	Serious	Not serious	Not serious	Not assessed ^d^	⨁◯◯◯ Very low
Gestational Diabetes Mellitus	10 [19,49,56,57,62,69,71,72,74,78]	Not serious	Serious	Not serious	Not serious	strongly suspected ^e^	⨁◯◯◯ Very low
Gestational Hypertension	3 [20,39,52]	Not serious	Serious	Not serious	Not serious	Not assessed ^d^	⨁◯◯◯ Very low
Preeclampsia	4 [20,39,75,76]	Not serious	Not serious	Not serious	Not serious	Not assessed ^d^	⨁⨁⨁◯ Moderate
Neonatal Outcomes							
Low Birth Weight	5 [40,44,48,53,73]	Not serious	Serious	Not serious	Not serious	Not assessed ^d^	⨁◯◯◯ Very low
Large for Gestational Age	3 [54,66,73]	Not serious	Serious	Not serious	Not serious	Not assessed ^d^	⨁◯◯◯ Very low
Preterm Birth	4 [48,55,83,85]	Not serious	Serious	Not serious	Not serious	Not assessed ^d^	⨁◯◯◯ Very low

^a^ Downgrade 1 level if I^2^ was 50% to 75%, and 2 levels if I^2^ was 75% to 100%. ^b^ No downgrade for indirectness because all studies directly measure the outcomes. ^c^ No downgrade for imprecision because of >2000 participants for each outcome. ^d^ No downgrade for publication bias, as publication bias could not be assessed due to lack of power for assessing funnel plot asymmetry and small study effects (<10 cohorts included in meta-analysis). ^e^ Downgrade 1 level for publication bias (*p* < 0.05).

## Data Availability

Not applicable.

## Supplementary Materials

The following supporting information can be downloaded at: https://www.mdpi.com/article/10.3390/nu14153242/s1, Table S1: PECOS acronym used in the design of the study; Table S2: Database search strategies; Table S3: reasons for exclusion of articles; Table S4: risk of bias of cohort studies; Table S5: risk of bias of cross-sectional studies; Table S6: risk of bias of case-control studies; Figure S1: Publication bias funnel graph for UPF consumption and Gestational Diabetes Mellitus risk.
