# Supplementary material for: Maternal Consumption of Ultra-Processed Foods-Rich Diet and Perinatal Outcomes: A Systematic Review and Meta-Analysis
Source: Nutrients. 2022 Aug 8;14(15):3242. doi: 10.3390/nu14153242 (PMC9370797; doi:10.3390/nu14153242)
Supplement: Supplementary file 1 [file nutrients-14-03242-s001.zip › 2. Table S1 PECOS acronym used in the design of the study.pdf]

Parameter	Criteria
**Participants**	Pregnant women
**Exposure**	Ultra-processed food consumption
**Comparisons**	-
**Outcomes**	Maternal and neonatal outcomes<br>(i.e. gestational hypertension and diabetes, gestational weight gain, birth size, prematurity)
**Study design**	Observational studies

Table S1: PECOS acronym used in the design of the study
