# Supplementary material for: Maternal Consumption of Ultra-Processed Foods-Rich Diet and Perinatal Outcomes: A Systematic Review and Meta-Analysis
Source: Nutrients. 2022 Aug 8;14(15):3242. doi: 10.3390/nu14153242 (PMC9370797; doi:10.3390/nu14153242)
Supplement: Supplementary file 1 [file nutrients-14-03242-s001.zip › 3. Table S2_ Database search strategies.pdf]

Table S2. Database search strategies for systematic review of the consumption of ultra-processed and unhealthy foods and maternal/neonatal outcomes

| DATABASE | SEARCH (date) |
|---|---|
| **MEDLINE** | ("pregnancy"[MeSH Terms] OR ("pregnancy"[MeSH Terms] OR "pregnancy"[All Fields] OR "pregnancies"[All Fields] OR "pregnancy s"[All Fields]) OR ("gestate"[All Fields] OR "gestated"[All Fields] OR "gestates"[All Fields] OR "gestating"[All Fields] OR "gestational"[All Fields] OR "gestations"[All Fields] OR "pregnancy"[MeSH Terms] OR "pregnancy"[All Fields] OR "gestation"[All Fields]) OR "pregnan*"[All Fields] OR "pregnant women"[MeSH Terms] OR "pregnant woman"[All Fields] OR "woman pregnant"[All Fields] OR "women pregnant"[All Fields] OR ("maternally"[All Fields] OR "maternities"[All Fields] OR "maternity"[All Fields] OR "mothers"[MeSH Terms] OR "mothers"[All Fields] OR "maternal"[All Fields]) OR ("antenatal"[All Fields] OR "antenatally"[All Fields]) OR "maternal exposure"[MeSH Terms]) AND ("fast foods"[MeSH Terms] OR "fast food"[All Fields] OR "Ultraprocessed food"[All Fields] OR "Ultraprocessed foods"[All Fields] OR "Ultra-processed food"[All Fields] OR "Ultra-processed foods"[All Fields] OR "Industrialized food"[All Fields] OR "Processed food"[All Fields] OR "Ready-to-eat meal"[All Fields] OR "ready-to-eat food"[All Fields] OR "Ready-Prepared Food"[All Fields] OR "non-home-prepared food"[All Fields] OR "Sodium intake"[All Fields] OR "salt intake"[All Fields] OR "salt food"[All Fields] OR "Salty food"[All Fields] OR "diet, high fat"[MeSH Terms] OR "high-fat diet"[All Fields] OR "High-fat food"[All Fields] OR "high-sucrose"[All Fields] OR "high-salt"[All Fields] OR "High sodium food"[All Fields] OR "highly processed foods"[All Fields] OR "refined food"[All Fields] OR "high sugar"[All Fields] OR "junk food"[All Fields] OR "sugar-sweetened beverages"[MeSH Terms] OR "sugar-sweetened beverages"[All Fields] OR "soft drink"[All Fields] OR "unhealthy eating"[All Fields] OR "unhealthy diet"[All Fields] OR "unhealthy nutrition"[All Fields] OR "Unhealthy dietary habits"[All Fields] OR "Unhealthy dietary patterns"[All Fields] OR "unhealthy dietary behaviors"[All Fields] OR "unhealthy food"[All Fields] OR "diet quality"[All Fields] OR "dietary energy density"[All Fields] OR "High energy density"[All Fields] OR "poor diet"[All Fields] OR "dietary quality"[All Fields] OR "processed meat"[All Fields] OR "diet, western"[MeSH Terms] OR "western diets"[All Fields]) AND ("Perinatal outcome"[All Fields] OR "perinatal outcomes"[All Fields] OR "Pregnancy outcomes"[All Fields] OR "pregnancy outcome"[All Fields] OR "outcome of pregnancy"[All Fields] OR "gestational weight gain"[MeSH Terms] OR "pregnancy weight gain"[All Fields] OR "birth outcomes"[All Fields] OR "birth outcome"[All Fields] OR "birth weight"[MeSH Terms] OR ("birth weight"[MeSH Terms] OR ("birth"[All Fields] AND "weight"[All Fields]) OR "birth weight"[All Fields] OR "birthweight"[All Fields] OR "birthweights"[All Fields]) OR "Neonatal weight"[All Fields] OR "Newborn weight"[All Fields] OR "birth size"[All Fields] OR "hypertension, pregnancy induced"[MeSH Terms] OR "hypertension, pregnancy induced"[MeSH Terms] OR "Hypertensive disorders"[All Fields] OR "Blood pressure"[All Fields] OR "Hypertension"[All Fields] OR "diabetes, gestational"[MeSH Terms] OR "glycemic outcomes"[All Fields] OR "premature birth"[MeSH Terms] OR "Preterm birth"[All Fields] OR "Preterm delivery"[All Fields] OR "prematurity"[All Fields] OR "fetal growth"[All Fields] OR "pregnancy complications"[MeSH Terms]) |
| **EMBASE** | (pregnancy OR pregnancies OR gestation OR pregnan* OR 'pregnant women' OR 'pregnant woman' OR 'woman, pregnant' OR 'women, pregnant' OR maternal OR antenatal OR 'maternal exposure') AND ('ultraprocessed food' OR 'ultraprocessed foods' OR 'ultra-processed food' OR 'ultra-processed foods' OR 'industrialized food' OR 'processed food' OR 'ready-to-eat meal' OR 'ready-to-eat food' OR 'ready-prepared food' OR 'fast food' OR 'non-home-prepared food' OR 'sodium intake' OR 'salt intake' OR 'salt food' OR 'salty food' OR 'high-fat food' OR 'high-fat diet' OR 'high-sucrose' OR 'high-salt' OR 'high sodium food' OR 'soft drink' OR 'highly processed foods' OR 'refined food' OR 'high sugar' OR 'junk food' OR 'sugar-sweetened beverage' OR 'sugar-sweetened beverages' OR 'unhealthy eating' OR 'unhealthy diet' OR 'unhealthy nutrition' OR 'unhealthy dietary habits' OR 'unhealthy dietary patterns' OR 'unhealthy dietary behaviors' OR 'unhealthy food' OR 'diet quality' OR 'dietary energy density' OR 'high energy density' OR 'poor diet' OR 'dietary quality' OR 'processed meat' OR 'western diet' OR snacks OR 'processing pattern' OR 'added sugar' OR 'refined grain') AND ('perinatal outcome' OR 'perinatal outcomes' OR 'gestational weight gain' OR 'pregnancy weight gain' OR 'birth outcomes' OR 'birth outcome' OR 'birth weight' OR 'hypertensive disorders' OR 'pregnancy-induced hypertension' OR 'gestational |

| | |
|---|---|
| | diabetes' OR 'blood pressure' OR hypertension OR 'gestational hypertension' OR 'preterm birth' OR 'preterm delivery' OR 'premature birth' OR prematurity OR 'fetal growth' OR 'pregnancy complications' OR 'pregnancy outcomes' OR 'pregnancy outcome' OR 'outcome of pregnancy' OR 'glycemic outcomes' OR 'birth size' OR birthweight OR 'neonatal weight' OR 'newborn weight' OR 'small-for-gestational age') |
| **LILACS** | (pregnancy OR pregnancies OR gestation OR pregnan* OR 'pregnant women' OR 'pregnant woman' OR 'woman, pregnant' OR 'women, pregnant' OR maternal OR antenatal OR 'maternal exposure') AND ('ultraprocessed food' OR 'ultraprocessed foods' OR 'ultra-processed food' OR 'ultra-processed foods' OR 'industrialized food' OR 'processed food' OR 'ready-to-eat meal' OR 'ready-to-eat food' OR 'ready-prepared food' OR 'fast food' OR 'non-home-prepared food' OR 'sodium intake' OR 'salt intake' OR 'salt food' OR 'salty food' OR 'high-fat food' OR 'high-fat diet' OR 'high-sucrose' OR 'high-salt' OR 'high sodium food' OR 'soft drink' OR 'highly processed foods' OR 'refined food' OR 'high sugar' OR 'junk food' OR 'sugar-sweetened beverage' OR 'sugar-sweetened beverages' OR 'unhealthy eating' OR 'unhealthy diet' OR 'unhealthy nutrition' OR 'unhealthy dietary habits' OR 'unhealthy dietary patterns' OR 'unhealthy dietary behaviors' OR 'unhealthy food' OR 'diet quality' OR 'dietary energy density' OR 'high energy density' OR 'poor diet' OR 'dietary quality' OR 'processed meat' OR 'western diet' OR snacks OR 'processing pattern' OR 'added sugar' OR 'refined grain') AND ('perinatal outcome' OR 'perinatal outcomes' OR 'gestational weight gain' OR 'pregnancy weight gain' OR 'birth outcomes' OR 'birth outcome' OR 'birth weight' OR 'hypertensive disorders' OR 'pregnancy-induced hypertension' OR 'gestational diabetes' OR 'blood pressure' OR hypertension OR 'gestational hypertension' OR 'preterm birth' OR 'preterm delivery' OR 'premature birth' OR prematurity OR 'fetal growth' OR 'pregnancy complications' OR 'pregnancy outcomes' OR 'pregnancy outcome' OR 'outcome of pregnancy' OR 'glycemic outcomes' OR 'birth size' OR birthweight OR 'neonatal weight' OR 'newborn weight' OR 'small-for-gestational age') |
| **SCOPUS** | ( ( TITLE-ABS-KEY ( pregnancy ) OR TITLE-ABS-KEY ( pregnancies ) OR TITLE-ABS-KEY ( gestation ) OR TITLE-ABS-KEY ( pregnan* ) OR TITLE-ABS-KEY ( pregnant AND women ) OR TITLE-ABS-KEY ( "Pregnant woman" ) OR TITLE-ABS-KEY ( "Woman, pregnant" ) OR TITLE-ABS-KEY ( "Women, pregnant" ) OR TITLE-ABS-KEY ( maternal ) OR TITLE-ABS-KEY ( antenatal ) OR TITLE-ABS-KEY ( "Maternal exposure" ) ) ) AND ( ( TITLE-ABS-KEY ( "Perinatal outcome" ) OR TITLE-ABS-KEY ( "perinatal outcomes" ) OR TITLE-ABS-KEY ( "gestational weight gain" ) OR TITLE-ABS-KEY ( "pregnancy weight gain" ) OR TITLE-ABS-KEY ( "birth outcomes" ) OR TITLE-ABS-KEY ( "birth outcome" ) OR TITLE-ABS-KEY ( "Birth weight" ) OR TITLE-ABS-KEY ( "Hypertensive disorders" ) OR TITLE-ABS-KEY ( "Pregnancy-Induced Hypertension" ) OR TITLE-ABS-KEY ( "Gestational diabetes" ) OR TITLE-ABS-KEY ( "Blood pressure" ) OR TITLE-ABS-KEY ( hypertension ) OR TITLE-ABS-KEY ( "Gestational Hypertension" ) OR TITLE-ABS-KEY ( "Preterm birth" ) OR TITLE-ABS-KEY ( "Preterm delivery" ) OR TITLE-ABS-KEY ( "Premature birth" ) OR TITLE-ABS-KEY ( prematurity ) OR TITLE-ABS-KEY ( "fetal growth" ) OR TITLE-ABS-KEY ( "Pregnancy complications" ) OR TITLE-ABS-KEY ( "Pregnancy outcomes" ) OR TITLE-ABS-KEY ( "pregnancy outcome" ) OR TITLE-ABS-KEY ( "outcome of pregnancy" ) OR TITLE-ABS-KEY ( "glycemic outcomes" ) OR TITLE-ABS-KEY ( "birth size" ) OR TITLE-ABS-KEY ( birthweight ) OR TITLE-ABS-KEY ( "Neonatal weight" ) OR TITLE-ABS-KEY ( "Newborn weight" ) OR TITLE-ABS-KEY ( "Small-for-gestational age" ) ) ) AND ( ( TITLE-ABS-KEY ( ultraprocessed AND food ) OR TITLE-ABS-KEY ( "Ultraprocessed foods" ) OR TITLE-ABS-KEY ( "Ultra-processed food" ) OR TITLE-ABS-KEY ( "Ultra-processed foods" ) OR TITLE-ABS-KEY ( "Industrialized food" ) OR TITLE-ABS-KEY ( "Processed food" ) OR TITLE-ABS-KEY ( "Ready-to-eat meal" ) OR TITLE-ABS-KEY ( "ready-to-eat food" ) OR TITLE-ABS-KEY ( "Ready-Prepared Food" ) OR TITLE-ABS-KEY ( "fast food" ) OR TITLE-ABS-KEY ( "non-home-prepared food" ) OR TITLE-ABS-KEY ( "Sodium intake" ) OR TITLE-ABS-KEY ( "salt intake" ) OR TITLE-ABS-KEY ( "salt food" ) OR TITLE-ABS-KEY ( "Salty food" ) OR TITLE-ABS-KEY ( "High-fat food" ) OR TITLE-ABS-KEY ( "high-fat diet" ) OR TITLE-ABS-KEY ( "high-sucrose" ) OR TITLE-ABS-KEY ( "high-salt" ) OR TITLE-ABS-KEY ( "High sodium food" ) OR TITLE-ABS-KEY ( "soft drink" ) OR TITLE-ABS-KEY ( "highly processed |

| | |
|---|---|
| | foods" ) OR TITLE-ABS-KEY ( "refined food" ) OR TITLE-ABS-KEY ( "high sugar" ) OR TITLE-ABS-KEY ( "junk food" ) OR TITLE-ABS-KEY ( "sugar-sweetened beverage" ) OR TITLE-ABS-KEY ( "sugar-sweetened beverages" ) OR TITLE-ABS-KEY ( "unhealthy eating" ) OR TITLE-ABS-KEY ( "unhealthy diet" ) OR TITLE-ABS-KEY ( "unhealthy nutrition" ) OR TITLE-ABS-KEY ( "Unhealthy dietary habits" ) OR TITLE-ABS-KEY ( "Unhealthy dietary patterns" ) OR TITLE-ABS-KEY ( "unhealthy dietary behaviors" ) OR TITLE-ABS-KEY ( "unhealthy food" ) OR TITLE-ABS-KEY ( "diet quality" ) OR TITLE-ABS-KEY ( "dietary energy density" ) OR TITLE-ABS-KEY ( "High energy density" ) OR TITLE-ABS-KEY ( "poor diet" ) OR TITLE-ABS-KEY ( "dietary quality" ) OR TITLE-ABS-KEY ( "processed meat" ) OR TITLE-ABS-KEY ( "western diet" ) OR TITLE-ABS-KEY ( snacks ) OR TITLE-ABS-KEY ( "processing pattern" ) OR TITLE-ABS-KEY ( "added sugar" ) OR TITLE-ABS-KEY ( "refined grain" ) ) ) |
| **WEB OF SCIENCE** | Pregnancy OR Pregnancies OR Gestation OR Pregnan* OR "Pregnant women" OR "Pregnant woman" OR "Woman, pregnant" OR "Women, pregnant" OR Maternal OR Antenatal OR "Maternal exposure" (All Fields) AND "Ultraprocessed food" OR "Ultraprocessed foods" OR "Ultra-processed food" OR "Ultra-processed foods" OR "Industrialized food" OR "Processed food" OR "Ready-to-eat meal" OR "ready-to-eat food" OR "Ready-Prepared Food" OR "fast food" OR "non-home-prepared food" OR "Sodium intake" OR "salt intake" OR "salt food" OR "Salty food" OR "High-fat food" OR "high-fat diet" OR "high-sucrose" OR "high-salt" OR "High sodium food" OR "soft drink" OR "highly processed foods" OR "refined food" OR "high sugar" OR "junk food" OR "sugar-sweetened beverage" OR "sugar-sweetened beverages" OR "unhealthy eating" OR "unhealthy diet" OR "unhealthy nutrition" OR "Unhealthy dietary habits" OR "Unhealthy dietary patterns" OR "unhealthy dietary behaviors" OR "unhealthy food" OR "diet quality" OR "dietary energy density" OR "High energy density" OR "poor diet" OR "dietary quality" OR "processed meat" OR "western diet" OR Snacks OR "processing pattern" OR "added sugar" OR "refined grain" (All Fields) AND "Perinatal outcome" OR "perinatal outcomes" OR "gestational weight gain" OR "pregnancy weight gain" OR "birth outcomes" OR "birth outcome" OR "Birth weight" OR "Hypertensive disorders" OR "Pregnancy-Induced Hypertension" OR "Gestational diabetes" OR "Blood pressure" OR hypertension OR "Gestational Hypertension" OR "Preterm birth" OR "Preterm delivery" OR "Premature birth" OR prematurity OR "fetal growth" OR "Pregnancy complications" OR "Pregnancy outcomes" OR "pregnancy outcome" OR "outcome of pregnancy" OR "glycemic outcomes" OR "birth size" OR Birthweight OR "Neonatal weight" OR "Newborn weight" OR "Small-for-gestational age" (All Fields) |
| **GOOGLE SCHOLAR** | Pregnancy OR "Pregnant women" OR "Pregnant woman" AND "Ultraprocessed food" OR "Ultra-processed foods" OR "Industrialized food" OR "Processed food" OR "Sodium intake" OR "salt intake" OR "High-fat food" OR "high-sucrose" OR "soft drink" OR "junk food" OR "sugar-sweetened beverage" OR "unhealthy diet" OR "Unhealthy dietary habits" OR "Unhealthy dietary patterns" OR "unhealthy dietary behaviors" OR "processed meat" OR "western diet" OR Snacks AND "Perinatal outcome" OR "gestational weight gain" OR "birth outcomes" OR "Birth weight" OR "Hypertensive disorders" OR "Pregnancy-Induced Hypertension" OR "Gestational diabetes" OR "Preterm birth" OR "Premature birth" OR "fetal growth" OR "Pregnancy complications" OR "birth size OR "Small-for-gestational age" |
| **ProQuest** | ab(Pregnancy OR Pregnancies OR Gestation OR Pregnan* OR "Pregnant women" OR "Pregnant woman" OR "Woman, pregnant" OR "Women, pregnant" OR Maternal OR Antenatal OR "Maternal exposure") AND ab("Ultraprocessed food" OR "Ultraprocessed foods" OR "Ultra-processed food" OR "Ultra-processed foods" OR "Industrialized food" OR "Processed food" OR "Ready-to-eat meal" OR "ready-to-eat food" OR "Ready-Prepared Food" OR "fast food" OR "non-home-prepared food" OR "Sodium intake" OR "salt intake" OR "salt food" OR "Salty food" OR "High-fat food" OR "high-fat diet" OR "high-sucrose" OR "high-salt" OR "High sodium food" OR "soft drink" OR "highly processed foods" OR "refined food" OR "high sugar" OR "junk food" OR "sugar-sweetened beverage" OR "sugar-sweetened beverages" OR "unhealthy eating" OR "unhealthy diet" OR "unhealthy nutrition" OR "Unhealthy dietary habits" OR "Unhealthy dietary patterns" OR "unhealthy dietary behaviors" OR "unhealthy food" OR "diet quality" OR "dietary energy density" OR "High |

| | |
|---|---|
| | energy density" OR "poor diet" OR "dietary quality" OR "processed meat" OR "western diet" OR Snacks OR "processing pattern" OR "added sugar" OR "refined grain") AND ab("Perinatal outcome" OR "perinatal outcomes" OR "gestational weight gain" OR "pregnancy weight gain" OR "birth outcomes" OR "birth outcome" OR "Birth weight" OR "Hypertensive disorders" OR "Pregnancy-Induced Hypertension" OR "Gestational diabetes" OR "Blood pressure" OR hypertension OR "Gestational Hypertension" OR "Preterm birth" OR "Preterm delivery" OR "Premature birth" OR prematurity OR "fetal growth" OR "Pregnancy complications" OR "Pregnancy outcomes" OR "pregnancy outcome" OR "outcome of pregnancy" OR "glycemic outcomes" OR "birth size" OR Birthweight OR "Neonatal weight" OR "Newborn weight" OR "Small-for-gestational age") |
