# Supplementary material for: Maternal Consumption of Ultra-Processed Foods-Rich Diet and Perinatal Outcomes: A Systematic Review and Meta-Analysis
Source: Nutrients. 2022 Aug 8;14(15):3242. doi: 10.3390/nu14153242 (PMC9370797; doi:10.3390/nu14153242)
Supplement: Supplementary file 1 [file nutrients-14-03242-s001.zip › 4. Table S3_Excluded articles and reasons for exclusion.pdf]

Table S3. Excluded articles and reasons for exclusion

Author, Year, Reference	Reason for exclusion
Wen et al., 2010[1]; Mahabamunuge et al., 2021[2]; Figueroa et al., 2020[3]; Menezes et al., 2019[4]; Martin et al., 2016[5]; Martin et al., 2015[6].	1
Chen et al., 2021[7]; Octavia et al., 2020[8]; Madzorera et al.,2020[9]; Abu-Saad et al.,2021[10]; Augustin, H., Winkvist, A. & Bärebring, 2020[11]; Yee et al., 2020[12]; Wrottesley et al.,[13]; Parker et al., 2019[14]; Gonzalez-Nahm et al.,2019[15]; Tahir et al., 2019[16]; Battarbee et al., 2019[17]; Emond et al., 2018[18]; Anand et al., 2017[19]; Crozier et al., 2017[20]; Gresham et al., 2016[21]; Zhang et al., 2014[22]; Deierlein et al., 2008[23]; Philipps et al., 1997[24]; Roustazadeh et al., 2021[25]; Trumpff et al., 2021[26]; Yan et al., 2020[27];Yong et al., 2020[28]; Dąbrowski, M. & Pawluś, D., 2017[29]; Gicevic, et al., 2018[30]; de Seymour et al., 2016[31]; Schoenaker et al., 2015[32]; Shin et al., 2015[33]; Gaskins et al., 2014[34]; Starling et al., 2017[35]; Chen et al., 2017[36]; Osorio-Yáñez et al., 2017[37]; Ameen et al., 2021[38]; da Mota Santana et al., 2021[39]; Yang et al., 2022[40].	2
Grundt et al., 2012[41]; Alabed et al., 2021[42]; Baxi and Shah, 2021[43]; Singh et al., 2021[44]; Yu et al., 2021[45].	3
Longmore et al., 2020[46]; Cummings et al., 2022 [47].	4
Englund-Ögge et al., 2012[48]; Halldorsson et al., 2010; [49]; Sartorelli et al., 2019 [50]; Arvizu Boy, 2019[51]; Leone et al., 2021[52].	5
Perez et al., 2021[53]; Nagel et al., 2020[54]; Whyte et al., 2020[55]; Leung et al., 2016[56]; Jarman et al., 2016[57]; Zhu et al., 2016[58]; Starling et al., 2016[59]; Tielemans et al., 2015[60]; Englund-Ogge et al., 2013[61]; Von et al., 2012[62]; Both et al., 2012 [63].	6
Chen et al., 2021[64]; Nascimento et al., 2017 [65]; Gillman et al., 2017 [66]; Loo et al., 2017[67]; Azad et al., 2016[68]; Von Ehrenstein et al., 2015[69]; Zhu et al., 2017[70]; Pinto et al., 2021[71]; Murrin et al., 2015[72]; Hu et al., 2020[73].	7
Akhmetova et al., 2010[74]; Yan et al., 2019[75]; Rajaei et al., 2019[76]; Taheri et al., 2018[77]; Sokup et al., 2010[78]; Sokup et al., 2010[79]; Li et al., 2015[80]; Samohýl et al., 2016[81]; Miranda, 2021[82]; wang et al., 2021[83].	8
Mizgier et al., 2021[84]; Brantsæter et al., 2014[85]; Phelan et al., [86]; Whyte, 2019[87].	9
Bursey; Watson, 2010[88]; Vecchia, 2010[89]; Moses; Brand-Miller, 2009[90].	10

(1) Studies do not evaluate UPF association with perinatal outcomes; (2) Studies do not evaluate UPF group consumption; (3) Studies do not report association data; (4) Participants had comorbidities at baseline (5) Same sample from previous included study; (6) Conference/magazine abstract; (7) Studies do not report perinatal outcomes; (8) No responses to contact attempts for additional information; (9) Study design; (10) Letters to editor/editorial.

References

[1] L. M. Wen, V. M. Flood, J. M. Simpson, C. Rissel, and L. A. Baur, "Dietary behaviours during pregnancy: Findings from first-time mothers in southwest Sydney, Australia," *Int. J. Behav. Nutr. Phys. Act.*, vol. 7, pp. 1–7, 2010, doi: 10.1186/1479-5868-7-13.

[2] J. Mahabamunuge *et al.*, "Association of sugar-sweetened beverage intake with maternal postpartum weight retention," *Public Health Nutr.*, vol. 24, no. 13, pp. 4196–4203, 2021, doi: 10.1017/S1368980020005169.

[3] R. Figueroa, J. A. Saltzman, A. Kang, F. N. Mini, K. K. Davison, and E. M. Taveras, "Antenatal dietary concordance among mothers and fathers and gestational weight gain: A longitudinal study," *BMC Public Health*, vol. 20, no. 1, pp. 1–10, 2020, doi: 10.1186/s12889-020-09182-7.

[4] C. C. Menezes *et al.*, "Soft drink consumption and periodontal status in pregnant women," *J. Periodontol.*, vol. 90, no. 2, pp. 159–166, 2019, doi: 10.1002/JPER.16-0388.

[5] C. L. Martin *et al.*, "Maternal Dietary Patterns are Associated with Lower Levels of Cardiometabolic Markers during Pregnancy," *Paediatr. Perinat. Epidemiol.*, vol. 30, no. 3, pp. 246–255, May 2016, doi: 10.1111/ppe.12279.

[6] C. L. Martin, "Association between maternal dietary patterns during pregnancy and early childhood growth outcomes," *ProQuest Diss. Theses*, p. 112, 2015, [Online]. Available: https://www.proquest.com/dissertations-theses/association-between-maternal-dietary-patterns/docview/1717894311/se-2?accountid=26646%0Ahttp://link.periodicos.capes.gov.br/sfxlcl41?url_ver=Z39.88-2004&rft_val_fmt=info:ofi/fmt:kev:mtx:dissertation&genre=diss.

[7] L. W. Chen *et al.*, "Associations of maternal dietary inflammatory potential and quality with offspring birth outcomes: An individual participant data pooled analysis of 7 European cohorts in the ALPHABET consortium," *PLoS Med.*, vol. 18, no. 1, pp. 1–22, 2021, doi: 10.1371/JOURNAL.PMED.1003491.

[8] L. Octavia *et al.*, "Associations of maternal diet quality with weight gain during pregnancy and obesity at three-year postpartum in Jakarta," *PLoS One*, vol. 15, no. 12 December 2020, pp. 1–16, 2020, doi: 10.1371/journal.pone.0244449.

[9] I. Madzorera *et al.*, "Maternal dietary diversity and dietary quality scores in relation to adverse birth outcomes in Tanzanian women," *Am. J. Clin. Nutr.*, vol. 112, no. 3, pp. 695–706, Aug. 2020, doi: 10.1093/ajcn/nqaa172.

[10] K. Abu-Saad, V. Kaufman-Shriqui, L. S. Freedman, I. Belmaker, and D. Fraser, "Preconceptional diet quality is associated with birth outcomes among low socioeconomic status minority women in a high-income country," *Eur. J. Nutr.*, vol. 60, no. 1, pp. 65–77, 2021, doi: 10.1007/s00394-020-02221-4.

[11] H. Augustin, A. Winkvist, and L. Bärebring, "Poor dietary quality is associated with low adherence to gestational weight gain recommendations among women in Sweden," *Nutrients*, vol. 12, no. 2, pp. 7–11, 2020, doi: 10.3390/nu12020317.

[12] L. M. Yee *et al.*, "Quality of periconceptional dietary intake and maternal and neonatal outcomes," *Am. J. Obstet. Gynecol.*, vol. 223, no. 1, pp. 121.e1-121.e8, 2020, doi: 10.1016/j.ajog.2020.01.042.

[13] S. V. Wrottesley, A. Prioreschi, S. H. Kehoe, K. A. Ward, and S. A. Norris, "A maternal 'mixed, high sugar' dietary pattern is associated with fetal growth," *Matern. Child Nutr.*, vol. 16, no. 2, pp. 1–11, 2020, doi: 10.1111/mcn.12912.

[14] H. W. Parker, A. Tovar, K. McCurdy, and M. Vadiveloo, "Associations between pre-pregnancy BMI, gestational weight gain, and prenatal diet quality in a national sample," *PLoS One*, vol. 14, no. 10, pp. 1–18, 2019, doi: 10.1371/journal.pone.0224034.

[15] S. Gonzalez-Nahm, C. Hoyo, T. Østbye, B. Neelon, C. Allen, and S. E. Benjamin-Neelon,

"Associations of maternal diet with infant adiposity at birth, 6 months and 12 months," *BMJ Open*, vol. 9, no. 9, pp. 1–7, 2019, doi: 10.1136/bmjopen-2019-030186.

[16] M. J. Tahir *et al.*, "Higher maternal diet quality during pregnancy and lactation is associated with lower infant weight-for-length, body fat percent, and fat mass in early postnatal life," *Nutrients*, vol. 11, no. 3, pp. 1–14, 2019, doi: 10.3390/nu11030632.

[17] A. N. Battarbee and L. M. Yee, "Association of Fast Food and Supermarket Density with Neonatal Outcomes of Pregnancies Affected by Gestational Diabetes," *Am. J. Perinatol.*, vol. 36, no. 13, pp. 1405–1411, Nov. 2019, doi: 10.1055/s-0038-1677478.

[18] J. A. Emond, M. R. Karagas, E. R. Baker, and D. Gilbert-Diamond, "Better diet quality during pregnancy is associated with a reduced likelihood of an infant born small for gestational age: An analysis of the prospective New Hampshire birth cohort study," *J. Nutr.*, vol. 148, no. 1, pp. 22–30, 2018, doi: 10.1093/jn/nxx005.

[19] S. S. Anand *et al.*, "Causes and consequences of gestational diabetes in South Asians living in Canada: results from a prospective cohort study," *C. Open*, vol. 5, no. 3, pp. E604–E611, 2017, doi: 10.9778/cmajo.20170027.

[20] S. R. Crozier, H. M. Inskip, K. M. Godfrey, C. Cooper, and S. M. Robinson, "Nausea and vomiting in early pregnancy: Effects on food intake and diet quality," *Matern. Child Nutr.*, vol. 13, no. 4, pp. 1–10, 2017, doi: 10.1111/mcn.12389.

[21] E. Gresham, C. E. Collins, G. D. Mishra, J. E. Byles, and A. J. Hure, "Diet quality before or during pregnancy and the relationship with pregnancy and birth outcomes: The Australian Longitudinal Study on Women's Health," *Public Health Nutr.*, vol. 19, no. 16, pp. 2975–2983, 2016, doi: 10.1017/S1368980016001245.

[22] C. Zhang *et al.*, "Adherence to healthy lifestyle and risk of gestational diabetes mellitus: Prospective cohort study," *BMJ*, vol. 349, no. September, pp. 1–11, 2014, doi: 10.1136/bmj.g5450.

[23] A. L. Deierlein, A. M. Siega-Riz, and A. Herring, "Dietary energy density but not glycemic load is associated with gestational weight gain," *Am. J. Clin. Nutr.*, vol. 88, no. 3, pp. 693–699, 2008, doi: 10.1093/ajcn/88.3.693.

[24] C. Philipps and N. E. Johnson, "The impact of quality of diet and other factors on birth weight of infants," *Am. J. Clin. Nutr.*, vol. 30, no. 2, pp. 215–225, 1977, doi: 10.1093/ajcn/30.2.215.

[25] A. Roustazadeh *et al.*, "A dietary pattern rich in fruits and dairy products is inversely associated to gestational diabetes: a case-control study in Iran," *BMC Endocr. Disord.*, vol. 21, no. 1, pp. 1–9, 2021, doi: 10.1186/s12902-021-00707-8.

[26] C. Trumpff *et al.*, "Added sugar intake during pregnancy: Fetal behavior, birth outcomes, and placental DNA methylation," *Dev. Psychobiol.*, vol. 63, no. 5, pp. 878–889, 2021, doi: 10.1002/dev.22088.

[27] H. Yan, S. Dang, Y. Zhang, and S. Luo, "Dietary patterns of Chinese women of childbearing age during pregnancy and their relationship to the neonatal birth weight," *Nutr. J.*, vol. 19, no. 1, p. 89, Dec. 2020, doi: 10.1186/s12937-020-00607-y.

[28] H. Y. Yong *et al.*, "The association between dietary patterns before and in early pregnancy and the risk of gestational diabetes mellitus (GDM): Data from the Malaysian SECOST cohort," *PLoS One*, vol. 15, no. 1, pp. 1–16, 2020, doi: 10.1371/journal.pone.0227246.

[29] M. Dąbrowski and D. Pawluś, "Association of food choices during pregnancy with gestational diabetes mellitus," *Clin. Diabetol.*, vol. 6, no. 4, pp. 131–135, 2017, doi: 10.5603/DK.2017.0022.

[30] S. Gicevic *et al.*, "Evaluating pre-pregnancy dietary diversity vs. dietary quality scores as predictors of gestational diabetes and hypertensive disorders of pregnancy," *PLoS One*, pp. 1–14, 2018, [Online]. Available: https://journals.plos.org/plosone/article?id=10.1371/journal.pone.0195103.

[31] J. de Seymour *et al.*, "Maternal dietary patterns and gestational diabetes mellitus in a multi-ethnic Asian cohort: The GUSTO study," *Nutrients*, vol. 8, no. 9, 2016, doi: 10.3390/nu8090574.

[32] D. A. J. M. Schoenaker, S. S. Soedamah-Muthu, L. K. Callaway, and G. D. Mishra, "Pre-pregnancy dietary patterns and risk of gestational diabetes mellitus: results from an Australian population-based prospective cohort study," *Diabetologia*, vol. 58, no. 12, pp. 2726–2735, 2015, doi: 10.1007/s00125-015-3742-1.

[33] D. Shin, K. W. Lee, and W. O. Song, "Dietary patterns during pregnancy are associated with risk of gestational diabetes mellitus," *Nutrients*, vol. 7, no. 11, pp. 9369–9382, 2015, doi: 10.3390/nu7115472.

[34] A. J. Gaskins *et al.*, "Prepregnancy dietary patterns and risk of pregnancy loss," *Am. J. Clin. Nutr.*, vol. 100, no. 4, pp. 1166–1172, 2014, doi: 10.3945/ajcn.114.083634.

[35] A. P. Starling, K. A. Sauder, J. L. Kaar, A. L. B. Shapiro, A. M. Siega-Riz, and D. Dabelea, "Maternal dietary patterns during pregnancy are associated with newborn body composition," *J. Nutr.*, vol. 147, no. 7, pp. 1334–1339, 2017, doi: 10.3945/jn.117.248948.

[36] L. W. Chen *et al.*, "Associations of maternal dietary patterns during pregnancy with offspring adiposity from birth until 54 months of age," *Nutrients*, vol. 9, no. 1, pp. 1–17, 2017, doi: 10.3390/nu9010002.

[37] C. Osorio-Yáñez *et al.*, "Maternal intake of fried foods and risk of gestational diabetes mellitus," *Ann. Epidemiol.*, vol. 27, no. 6, pp. 384-390.e1, Jun. 2017, doi: 10.1016/j.annepidem.2017.05.006.

[38] W. A. Ameen and K. J. Salma, "Maternal risk factors for hypertensive disorders during pregnancy," *Curr. Pediatr. Res.*, vol. 25, no. 9, pp. 961–967, 2021.

[39] J. da Mota Santana *et al.*, "Associations between Maternal Dietary Patterns and Infant Birth Weight in the NISAMI Cohort: A Structural Equation Modeling Analysis," *Nutrients*, vol. 13, no. 11, p. 4054, Nov. 2021, doi: 10.3390/nu13114054.

[40] J. Yang *et al.*, "Dietary diversity and diet quality with gestational weight gain and adverse birth outcomes, results from a prospective pregnancy cohort study in urban Tanzania," *Matern. Child Nutr.*, vol. 18, no. 2, Apr. 2022, doi: 10.1111/mcn.13300.

[41] J. H. Grundt, J. Nakling, G. E. Eide, and T. Markestad, "Possible relation between maternal consumption of added sugar and sugar-sweetened beverages and birth weight - Time trends in a population," *BMC Public Health*, vol. 12, no. 1, 2012, doi: 10.1186/1471-2458-12-901.

[42] A. A. A. Alabed, E. A. Aljrbi, A. Y. Al-Mahdi, R. Abdulsalam, A. R. Al-Maleki, and H. F. Ghazi, "Impacts of dietary supplements and nutrient-rich food for pregnant women on birth weight in Sugh El-Chmis /Alkhoms – Libya," *Malaysian J. Public Heal. Med.*, vol. 21, no. 2, pp. 426–433, 2021, doi: 10.37268/mjphm/vol.21/no.2/art.1114.

[43] B. Baxi and J. Shah, "Association of Pre-Pregnancy Body Mass Index and Gestational Weight Gain with Preterm Delivery in Pregnant Women," in *Current Topics in Caesarean Section*, IntechOpen, 2021.

[44] D. Singh *et al.*, "Effects of diet on hypertensive disorders during pregnancy: A cross-sectional study from a teaching hospital," *J. Fam. Med. Prim. Care*, vol. 10, no. 9, p. 3268, 2021, doi: 10.4103/jfmpc.jfmpc_96_21.

[45] Z. M. Yu *et al.*, "Predictors of Gestational Weight Gain Examined As a Continuous Outcome: A Prospective Analysis," *J. Women's Heal.*, vol. 30, no. 7, pp. 1006–1015, 2021, doi: 10.1089/jwh.2020.8799.

[46] D. K. Longmore *et al.*, "Social and economic factors, maternal behaviours in pregnancy and neonatal adiposity in the PANDORA cohort," *Diabetes Res. Clin. Pract.*, vol. 161, p. 108028, 2020, doi: 10.1016/j.diabres.2020.108028.

[47] J. R. Cummings, L. M. Lipsky, C. Schwedhelm, A. Liu, and T. R. Nansel, "Associations of ultra-processed food intake with maternal weight change and cardiometabolic health and infant growth.," *Int. J. Behav. Nutr. Phys. Act.*, vol. 19, no. 1, p. 61, May 2022, doi: 10.1186/s12966-022-01298-w.

[48] L. Englund-Ögge *et al.*, "Association between intake of artificially sweetened and sugar-

sweetened beverages and preterm delivery: A large prospective cohort study," *Am. J. Clin. Nutr.*, vol. 96, no. 3, pp. 552–559, 2012, doi: 10.3945/ajcn.111.031567.

[49] T. I. Halldorsson, M. Strøm, S. B. Petersen, and S. F. Olsen, "Intake of artificially sweetened soft drinks and risk of preterm delivery: A prospective cohort study in 59,334 Danish pregnant women," *Am. J. Clin. Nutr.*, vol. 92, no. 3, pp. 626–633, 2010, doi: 10.3945/ajcn.2009.28968.

[50] D. S. Sartorelli, L. C. Crivellenti, D. C. C. Zuccolotto, and L. J. Franco, "Relationship between minimally and ultra-processed food intake during pregnancy with obesity and gestational diabetes mellitus," *Cad. Saude Publica*, vol. 35, no. 4, pp. 1–10, 2019, doi: 10.1590/0102-311X00049318.

[51] M. Arvizu Boy, "Dietary Patterns and Hypertensive Disorders of Pregnancy," *Dr. Diss. ProQuest Diss. Theses*, p. 127, 2019, [Online]. Available: https://www.proquest.com/dissertations-theses/dietary-patterns-hypertensive-disorders-pregnancy/docview/2457693480/se-2?accountid=26646%0Ahttp://link.periodicos.capes.gov.br/sfxlcl41?url_ver=Z39.88-2004&rft_val_fmt=info:ofi/fmt:kev:mtx:dissertation&genre=.

[52] A. Leone, M. Á. Martínez-González, W. Craig, U. Fresán, C. Gómez-Donoso, and M. Bes-Rastrollo, "Pre-gestational consumption of ultra-processed foods and risk of gestational diabetes in a mediterranean cohort. The SUN project," *Nutrients*, vol. 13, no. 7, Jul. 2021, doi: 10.3390/nu13072202.

[53] M. Perez *et al.*, "756 Consumption of sugar sweetened and artificially sweetened beverages and pregnancy outcomes," *Am. J. Obstet. Gynecol.*, vol. 224, no. 2, pp. S473–S474, 2021, doi: 10.1016/j.ajog.2020.12.779.

[54] E. Nagel, E; Johnson, K; Foster, L; Duncan Smith, K; Kharbarda, E; Fields, D; Demerath, "Maternal added sugar intake is associated with infant adiposity and weight status at 6 months," *Obesity*, vol. 28, no. S2, pp. 40–187, Nov. 2020, doi: 10.1002/oby.23063.

[55] D. Whyte, K; Contento, I; Pi-Sunyer, X; Gallagher, "Oral Abstracts: Effect of ultra-processed food intake on excessive gestational weight gain for mothers with obesity," *Obesity*, vol. 28, no. S2, pp. 5–39, Nov. 2020, doi: 10.1002/oby.23057.

[56] B. A. Leung, C W; Epel, E S; Bush, N R; Coleman-Phox, K; Adler, N E; Laraia, "Maternal diet quality during pregnancy and fetal growth outcomes: A pilot study of lower-income pregnant women," *FASEB J.*, vol. 30, no. 0, 2016.

[57] P. J. Jarman, M; Bell, R C; Robson, "Conference Abstract: Dietary patterns and gestational weight gain in the alberta pregnancy outcomes and nutrition study," *FASEB J.*, vol. 30, 2016, [Online]. Available: https://www.embase.com/records?subaction=viewrecord&id=L72320795.

[58] C. Zhu, Y; Olsen, S F; Mendola, P; Hinkle, S N; Zhang, "Conference Abstract: Are you what your mother ate: Maternal sugar-sweetened beverages intake in pregnancy and offspring growth and obesity through age 7 years," *Diabetes*, vol. 65, no. Supplement_1, pp. A360–A431, Jun. 2016, doi: 10.2337/db16-1375-1656.

[59] D. Starling, A P; Kaar, J L; Sauder, K A; Shapiro, A L B; Dabelea, "Conference Abstract: Maternal dietary patterns during pregnancy and gestational weight gain: The healthy start study," *Diabetes*, vol. 65, no. Supplement_1, pp. A360–A431, Jun. 2016, doi: 10.2337/db16-1375-1656.

[60] J. C. Tielemans, M J; Erler, N S; Leermakers, E T M; Van Den Broek, M; Jaddoe, V W V; Steegers, E A P; Franco, O H; Kiefte-de Jong, "Dietary patterns during pregnancy and gestational weight gain: The generation R study," *Eur. J. Epidemiol.*, vol. 30, no. 8, pp. 709–1001, Aug. 2015, doi: 10.1007/s10654-015-0072-z.

[61] E.-O. L., J. B., B. A.-L., and S. V., "Scientific Abstract: A healthy pattern characterized by high intake of vegetables, fruits, and vegetable oils is associated with reduced risk of preterm delivery; results from a cohort study of 60,761 pregnant norwegian women," *Reprod. Sci.*, vol. 20, no. 3_suppl, pp. 61A-341A, Mar. 2013, doi: 10.1177/1933719113482088.

[62] O. Von Ehrenstein, K. Hoggatt, M. Wilhelm, and B. Ritz, "Prenatal fast food consumption and

risks for preterm birth in the urban environment of los angeles," *Epidemiology*, vol. 23, p. 1, Sep. 2012, doi: 10.1097/01.ede.0000417109.18791.db.
[63] M. I. Both *et al.*, "Scientific Abstract: The carbohydrate-rich dietary pattern used during the periconceptional period is associated with first trimester growth: The generation r study," *Reprod. Sci.*, vol. 19, no. 3_suppl, pp. 71A-404A, Mar. 2012, doi: 10.1177/1933719112442493.
[64] L. W. Chen *et al.*, "Maternal dietary quality, inflammatory potential and childhood adiposity: an individual participant data pooled analysis of seven European cohorts in the ALPHABET consortium," *BMC Med.*, vol. 19, no. 1, pp. 1–14, 2021, doi: 10.1186/s12916-021-01908-7.
[65] J. X. P. T. Nascimento *et al.*, "The First 1000 Days of Life Factors Associated with 'childhood Asthma Symptoms': Brisa Cohort, Brazil," *Sci. Rep.*, vol. 7, no. 1, pp. 1–12, 2017, doi: 10.1038/s41598-017-16295-4.
[66] M. W. Gillman, S. L. Rifas-Shiman, S. Fernandez-Barres, K. Kleinman, E. M. Taveras, and E. Oken, "Beverage intake during pregnancy and childhood adiposity," *Pediatrics*, vol. 140, no. 2, 2017, doi: 10.1542/peds.2017-0031.
[67] E. X. L. Loo *et al.*, "Effect of Maternal Dietary Patterns during Pregnancy on Self-Reported Allergic Diseases in the First 3 Years of Life: Results from the GUSTO Study," *Int. Arch. Allergy Immunol.*, vol. 173, no. 2, pp. 105–113, 2017, doi: 10.1159/000475497.
[68] M. B. Azad *et al.*, "Association between artificially sweetened beverage consumption during pregnancy and infant body mass index," *JAMA Pediatr.*, vol. 170, no. 7, pp. 662–670, 2016, doi: 10.1001/jamapediatrics.2016.0301.
[69] O. S. von Ehrenstein, H. Aralis, M. E. S. Flores, and B. Ritz, "Fast food consumption in pregnancy and subsequent asthma symptoms in young children," *Pediatr. Allergy Immunol.*, vol. 26, no. 6, pp. 571–577, 2015, doi: 10.1111/pai.12433.
[70] Y. Zhu *et al.*, "Maternal dietary intakes of refined grains during pregnancy and growth through the first 7 y of life among children born to women with gestational diabetes," *Am. J. Clin. Nutr.*, vol. 106, no. 1, pp. 96–104, 2017, doi: 10.3945/ajcn.116.136291.
[71] D. A. S. Pinto *et al.*, "High sugar content and body mass index: Modelling pathways around the first 1000 d of life, BRISA cohort," *Public Health Nutr.*, vol. 24, no. 15, pp. 4997–5005, 2021, doi: 10.1017/S136898002000525X.
[72] C. M Murrin, M. M Heinen, and C. C Kelleher, "Are Dietary Patterns of Mothers during Pregnancy Related to Childrens Weight Status? Evidence from the Lifeways Cross-Generational Cohort Study," *AIMS Public Heal.*, vol. 2, no. 3, pp. 274–296, 2015, doi: 10.3934/publichealth.2015.3.274.
[73] Z. Hu *et al.*, "Effects of maternal dietary patterns during pregnancy on early childhood growth trajectories and obesity risk: The CANDLE study," *Nutrients*, vol. 12, no. 2, pp. 1–13, 2020, doi: 10.3390/nu12020465.
[74] E. S. Akhmetova and V. A. Mudrov, "Diet Correction as the method of prevention of gestational diabetes mellitus," *Profil. meditsina*, vol. 23, no. 5, p. 50, 2020, doi: 10.17116/profmed20202305150.
[75] H. Yan, X. Y. Wu, S. N. Dang, Y. D. Zhang, and S. Y. Luo, "Study on the association of dietary patterns of Shaanxi women of childbearing age during pregnancy with adverse pregnancy outcomes from 2010 to 2012," *Zhonghua Yu Fang Yi Xue Za Zhi*, vol. 53, no. 8, pp. 829–834, Aug. 2019, doi: 10.3760/cma.j.issn.0253-9624.2019.08.006.
[76] R. M., V. Y., N. R., and D. A., "Relationship between maternal cardiovascular diseases risk factors during pregnancy and low birth weight in neonates by case-control method," *Iran. J. Obstet. Gynecol. Infertil.*, vol. 22, no. 6, pp. 65–74, 2019, doi: 10.22038/ijogi.2019.13745.
[77] S. A. Taheri, A. H. R. Ahmadi, M. Javadi, and A. Barikani, "Comparison of dietary patterns during pregnancy in the mothers of the infants with low birth weight and normal weight," *Iran. J. Obstet. Gynecol. Infertil.*, vol. 21, no. 1, pp. 80–89, 2018, doi: 10.22038/ijogi.2018.10585.
[78] A. Sokup, M. Mioduszewska, A. Bąk, and R. Kotzbach, "Unhealthy eating habits precede

gestational diabetes mellitus in Polish women Part I: Evaluation of frequency, regularity of consumed meals and consumed snacks, bread, sweets, fruit and vegetables. Eating habits and gestational diabetes," *Diabetol. Dosw. i Klin.*, vol. 10, no. 1, pp. 17–22, 2010.

[79] A. Sokup, M. Mioduszewska, A. Bąk, and R. Kotzbach, "Unhealthy eating habits precede gestational diabetes in Polish women Part II: Evaluation of the consumption of meat, fish, fat and ways of food preparation. Eating habits, food preparation and gestational diabetes," *Diabetol. Dosw. i Klin.*, vol. 10, no. 1, pp. 23–27, 2010.

[80] Q. Li, X. Y. Wu, S. Ang, M. Xie, and L. Shi, "Association of dietary habits with gestational diabetes mellitus among Cantonese women," *J. South. Med. Univ.*, vol. 35, no. 5, pp. 772–776, 2015.

[81] M. Samohýl *et al.*, "Impact of nutrition and the mother's lifestyle risk factors on the birth weight and birth length of newborns'," *Hygiena*, vol. 61, no. 2, pp. 48–55, 2016, doi: 10.21101/hygiena.a1384.

[82] C. Miranda, "Consumo de alimentos ultraprocessados na gestação: influência nas medidas antropométricas do bebê," Universidade Federal de Minas Gerais, 2021.

[83] N. Wang, Z. Deng, Y. Ding, Y. Liu, W. Hu, and G. He, "Effect of dietary patterns during pregnancy on the risk of developing gestational diabetes mellitus," *Chinese J. Diabetes Mellit.*, vol. 13, no. 9, pp. 865–871, 2021, doi: 10.3760/cma.j.cn115791-20210208-00081.

[84] M. Mizgier, G. Jarzabek-Bielecka, and K. Mruczyk, "Maternal diet and gestational diabetes mellitus development," *J. Matern. Neonatal Med.*, vol. 34, no. 1, pp. 77–86, Jan. 2021, doi: 10.1080/14767058.2019.1598364.

[85] A. L. Brantsæter *et al.*, "Diet matters, particularly in pregnancy – Results from MoBa studies of maternal diet and pregnancy outcomes," *Nor. Epidemiol.*, vol. 24, no. 1–2, pp. 63–77, 2014, doi: 10.5324/nje.v24i1-2.1805.

[86] S. Phelan *et al.*, "Maternal behaviors during pregnancy impact offspring obesity risk," *Exp. Diabetes Res.*, vol. 2011, 2011, doi: 10.1155/2011/985139.

[87] K. Whyte, "Investigating Associations between Consumption of Unprocessed and Ultra Processed Foods and Maternal and Neonatal Health Outcomes—Secondary Outcomes of LIFT Trial," Columbia University, 2019.

[88] R. G. Bursey and M. L. Watson, "Intake of artificially sweetened soft drinks and risk of preterm delivery," *Am. J. Clin. Nutr.*, vol. 92, no. 5, pp. 1277–1278, Nov. 2010, doi: 10.3945/ajcn.110.000984.

[89] C. La Vecchia, "Intake of artificially sweetened soft drinks and risk of preterm delivery," *Am. J. Clin. Nutr.*, vol. 92, no. 6, pp. 1540–1540, Dec. 2010, doi: 10.3945/ajcn.110.003566.

[90] R. G. Moses and J. C. Brand-Miller, "Dietary Risk Factors for Gestational Diabetes Mellitus," *Diabetes Care*, vol. 32, no. 12, pp. 2314–2315, Dec. 2009, doi: 10.2337/dc09-1640.
