# Supplementary material for: Maternal Consumption of Ultra-Processed Foods-Rich Diet and Perinatal Outcomes: A Systematic Review and Meta-Analysis
Source: Nutrients. 2022 Aug 8;14(15):3242. doi: 10.3390/nu14153242 (PMC9370797; doi:10.3390/nu14153242)
Supplement: Supplementary file 1 [file nutrients-14-03242-s001.zip › 5. Tables S4_S5_S6.pdf]

Table S4. Risk of bias for each individual study assessed by Joanna Briggs Institute critical appraisal checklist for cohort studies.

**Studies**	**Criteria**										
	**1***	**2***	**3***	**4***	**5***	**6***	**7***	**8***	**9***	**10***	**11***
Alves-Santos et al., 2019	Y	Y	Y	Y	Y	Y	Y	Y	Y	Y	Y
Ancira-Moreno et al., 2020	Y	Y	Y	Y	Y	Y	U	Y	Y	U	Y
Angali, Shahri & Borazjani, 2020	Y	Y	Y	Y	Y	Y	Y	Y	Y	N	Y
Barbosa et al., 2021	Y	Y	Y	Y	Y	U	N	Y	Y	N	Y
Bärebring et al., 2016	NA	Y	Y	Y	Y	Y	Y	N	Y	Y	Y
Baskin et al., 2015	Y	Y	Y	Y	Y	U	Y	Y	Y	Y	N
Borgen et al., 2012	Y	Y	Y	Y	Y	Y	Y	Y	Y	Y	Y
Brantsæter et al., 2009	Y	Y	Y	Y	Y	Y	Y	Y	Y	U	Y
Chen et al., 2009	Y	Y	Y	Y	Y	Y	N	Y	Y	U	Y
Coelho et al, 2015	N	Y	N	Y	Y	Y	N	U	U	U	Y
Dale et al, 2019	NA	Y	Y	Y	Y	Y	Y	Y	Y	Y	Y
Dominguez et al., 2014	Y	Y	Y	Y	Y	Y	Y	Y	Y	Y	Y
Donazar-Ezcurra et al., 2017	Y	Y	Y	Y	Y	Y	Y	Y	Y	Y	Y
Donazar-Ezcurra et al., 2017	Y	Y	Y	Y	Y	Y	Y	Y	Y	Y	Y
Englund-Ögge et al., 2014	Y	Y	Y	Y	Y	Y	Y	Y	U	U	Y
Englund-Ögge et al., 2014	Y	Y	Y	Y	Y	Y	Y	Y	Y	U	Y
Gomes et al, 2020	Y	Y	Y	Y	Y	Y	Y	Y	Y	Y	Y
Grundt et al., 2016	Y	Y	Y	Y	Y	Y	Y	Y	Y	Y	Y
Günther et al., 2019	Y	Y	Y	Y	Y	Y	Y	Y	Y	Y	Y
Hajianfar et al., 2018	Y	Y	Y	Y	Y	Y	Y	Y	Y	Y	Y
Hajianfar et al., 2018	Y	Y	Y	Y	Y	Y	Y	Y	Y	Y	Y
Hirko et al, 2020	Y	Y	Y	Y	Y	U	Y	Y	Y	Y	Y
Ikem et al., 2019	Y	Y	Y	Y	Y	Y	Y	Y	Y	Y	Y
Itani et al., 2020	Y	Y	Y	Y	Y	Y	Y	Y	Y	Y	Y
Ker et al., 2021	N	Y	N	Y	Y	U	Y	Y	Y	NA	Y
Lamyian et al., 2017	Y	Y	Y	Y	Y	Y	Y	Y	Y	Y	Y
Marí-Sanchiz et al., 2017	Y	Y	Y	Y	Y	Y	Y	Y	Y	Y	Y
Martin et al., 2016	Y	Y	Y	Y	Y	Y	N	Y	N	N	Y
Martin et al., 2015	Y	Y	N	Y	Y	Y	Y	Y	Y	Y	Y
Maugeri et al., 2019	Y	Y	Y	Y	Y	Y	Y	Y	Y	Y	Y
Mikeš et al., 2021	Y	Y	Y	Y	Y	Y	Y	N	Y	U	Y
Mitku et al., 2020	Y	Y	Y	Y	Y	Y	Y	Y	Y	Y	Y
Nascimento et al., 2016	Y	Y	Y	Y	Y	Y	Y	N	Y	Y	Y
Nicoli et al., 2021	U	Y	Y	Y	Y	U	Y	N	U	U	Y
Okubo et al, 2012	Y	Y	Y	Y	Y	Y	Y	Y	Y	Y	Y
Rasmussen et al., 2014	Y	Y	Y	Y	Y	Y	Y	Y	Y	Y	Y
Rohatgi et al., 2017	NA	NA	Y	Y	Y	Y	Y	Y	Y	U	Y
Schimidt et al., 2020	Y	Y	Y	Y	Y	Y	Y	Y	Y	Y	Y
Tamada et al., 2021	Y	Y	Y	Y	Y	Y	N	Y	Y	Y	Y
Teixeira et al., 2020	Y	Y	Y	Y	Y	Y	Y	Y	Y	Y	Y
Tielemans et al., 2015	Y	Y	Y	Y	Y	U	Y	Y	Y	Y	Y
Uusitalo et al., 2009	Y	Y	Y	Y	Y	Y	Y	N	Y	Y	Y
Wen et al., 2013	Y	Y	Y	Y	Y	Y	N	Y	Y	U	Y
Wrottesley, Pisa & Norris, 2017	Y	Y	Y	Y	Y	Y	Y	Y	Y	Y	Y
Yong et al.., 2021	Y	Y	Y	Y	Y	Y	Y	Y	Y	NA	Y
Zhang et al., 2006	Y	Y	Y	Y	Y	Y	Y	U	Y	Y	Y
Zhu et al., 2017	Y	Y	Y	Y	Y	Y	Y	Y	Y	Y	Y

Y = Yes, N = No, U = Unclear, NA = Not applicable

1* The two groups were similar and recruited from the same population.

2* The exposures were measured similarly to assign people to both exposed and unexposed groups.

3* The exposure was measured in a valid and reliable way.

4* The confounding factors were identified.

5* Strategies to deal with confounding factors were stated.

6* The groups/participants were free of the outcome at the start of the study (or at the moment of exposure).
7* The outcomes were measured in a valid and reliable way.
8* The follow up time was reported and sufficient to be long enough for outcomes to occur.
9* The follow up was complete, and if not, the reasons to loss to follow up were described and explored.
10* Strategies to address incomplete follow up were utilized.
11* Appropriate statistical analysis.

Table S5. Risk of bias for each individual study assessed by Joanna Briggs Institute critical appraisal checklist for cross-sectional studies.

Studies	Criteria							
	1*	2*	3*	4*	5*	6*	7*	8*
Ferreira et al., 2022	Y	Y	Y	Y	Y	Y	N	Y
Garay et al, 2019	Y	Y	Y	Y	Y	Y	Y	Y
Grieger, et al., 2014	Y	Y	Y	Y	Y	Y	Y	Y
Loy, Marhazlina & Jan, 2013	Y	Y	Y	Y	Y	Y	Y	Y
Liu et al., 2021	N	Y	Y	Y	Y	Y	N	Y
Marquez, 2012	Y	Y	Y	Y	Y	Y	Y	Y
Rodrigues, Azeredo & Silva, 2020	Y	Y	Y	Y	Y	Y	Y	Y
Zareei et al, 2019	Y	Y	Y	Y	Y	Y	Y	Y
Zuccolotto et al, 2019	Y	Y	Y	Y	Y	Y	Y	Y

Y = Yes, N = No, U = Unclear, NA = Not applicable
1* Criteria for inclusion in the sample clearly defined.
2* Study subjects and the setting described in detail.
3* Exposure measured in a valid and reliable way.
4* Objective, standard criteria used for measurement of the condition.
5* Confounding factors identified.
6* Strategies to deal with confounding factors stated.
7* Outcomes measured in a valid way.
8* Appropriate statistical analysis.

Table S6. Risk of bias for each individual study assessed by Joanna Briggs Institute critical appraisal checklist for case-control studies.

Studies	Criteria									
	1*	2*	3*	4*	5*	6*	7*	8*	9*	10*
Abbasi et al., 2019	Y	Y	Y	Y	Y	Y	Y	Y	Y	Y
Amezcua-Prieto et al, 2019	Y	Y	Y	Y	Y	Y	Y	Y	U	Y
Asadi et al., 2019	Y	Y	Y	Y	Y	Y	Y	Y	Y	Y
Chen et al., 2020	Y	Y	Y	Y	Y	Y	Y	Y	Y	Y
Sedaghat et al, 2017	Y	Y	Y	Y	Y	Y	Y	Y	Y	Y

Y = Yes, N = No, U = Unclear, NA = Not applicable
1* Groups comparable other than the presence of disease in cases or the absence of disease in controls.
2* Cases and controls matched appropriately.
3* Same criteria used for identification of cases and controls.
4* Exposure measured in a standard, valid and reliable way.
5* Exposure measured in the same way for cases and controls.
6* Confounding factors identified
7* Strategies to deal with confounding factors stated.
8* Outcomes assessed in a standard, valid and reliable way for cases and controls.
9* Exposure period of interest long enough to be meaningful.
10* Appropriate statistical analysis.
