# Supplementary material for: Maternal Consumption of Ultra-Processed Foods-Rich Diet and Perinatal Outcomes: A Systematic Review and Meta-Analysis
Source: Nutrients. 2022 Aug 8;14(15):3242. doi: 10.3390/nu14153242 (PMC9370797; doi:10.3390/nu14153242)
Supplement: Supplementary file 1 [file nutrients-14-03242-s001.zip › 6. Supplementary figure S1.pdf]

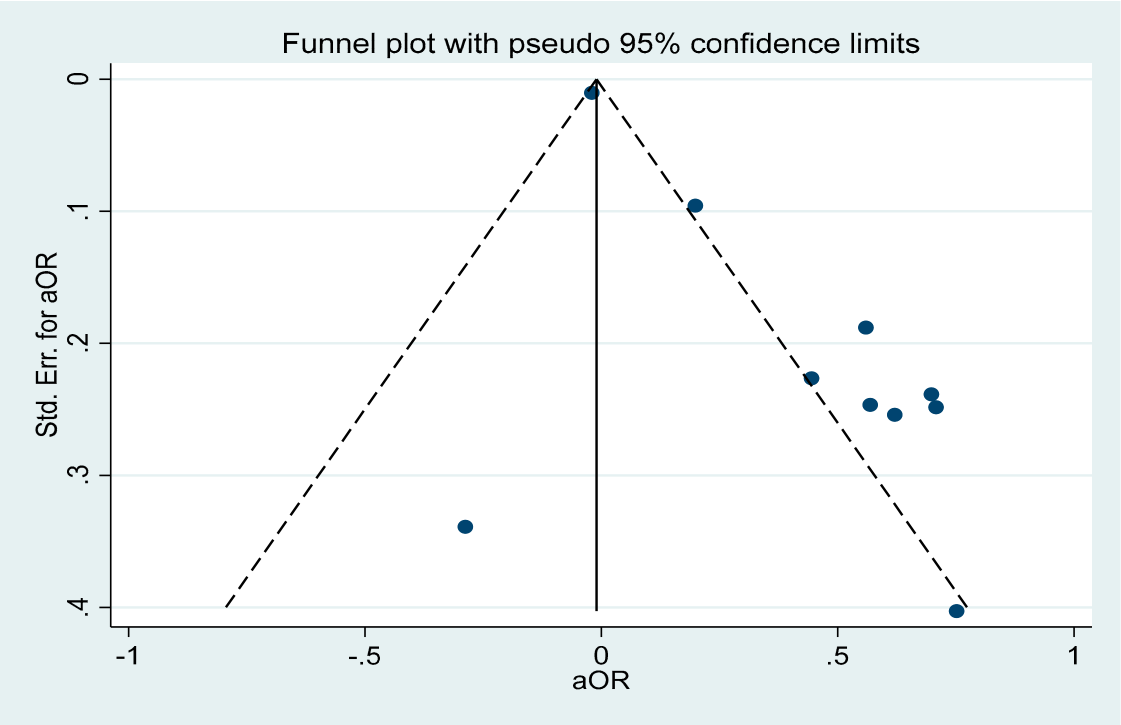

Supplementary figure S1. Publication bias funnel graph for UPF consumption and Gestational Diabetes Mellitus risk. Asymmetry was observed and confirmed by Egger test (p=0,001).
